# MAR‐Mediated transgene integration into permissive chromatin and increased expression by recombination pathway engineering

**DOI:** 10.1002/bit.26086

**Published:** 2016-10-03

**Authors:** Kaja Kostyrko, Samuel Neuenschwander, Thomas Junier, Alexandre Regamey, Christian Iseli, Emanuel Schmid‐Siegert, Sandra Bosshard, Stefano Majocchi, Valérie Le Fourn, Pierre‐Alain Girod, Ioannis Xenarios, Nicolas Mermod

**Affiliations:** ^1^ Department of Fundamental MicrobiologyInstitute of BiotechnologyUniversity of Lausanne, and Center for Biotechnology UNIL‐EPFLLausanneSwitzerland; ^2^ Swiss Institute of BioinformaticsLausanneSwitzerland; ^3^ Selexis SAGenevaSwitzerland

**Keywords:** DNA recombination, microhomology‐mediated end‐joining, Chinese hamster ovary cells, recombinant protein expression, immunoglobulin production

## Abstract

Untargeted plasmid integration into mammalian cell genomes remains a poorly understood and inefficient process. The formation of plasmid concatemers and their genomic integration has been ascribed either to non‐homologous end‐joining (NHEJ) or homologous recombination (HR) DNA repair pathways. However, a direct involvement of these pathways has remained unclear. Here, we show that the silencing of many HR factors enhanced plasmid concatemer formation and stable expression of the gene of interest in Chinese hamster ovary (CHO) cells, while the inhibition of NHEJ had no effect. However, genomic integration was decreased by the silencing of specific HR components, such as Rad51, and DNA synthesis‐dependent microhomology‐mediated end‐joining (SD‐MMEJ) activities. Genome‐wide analysis of the integration loci and junction sequences validated the prevalent use of the SD‐MMEJ pathway for transgene integration close to cellular genes, an effect shared with matrix attachment region (MAR) DNA elements that stimulate plasmid integration and expression. Overall, we conclude that SD‐MMEJ is the main mechanism driving the illegitimate genomic integration of foreign DNA in CHO cells, and we provide a recombination engineering approach that increases transgene integration and recombinant protein expression in these cells. Biotechnol. Bioeng. 2017;114: 384–396. © 2016 The Authors. *Biotechnology and Bioengineering* published by Wiley Periodicals, Inc.

## Introduction

Spontaneous integration of non‐viral DNA vectors into the genome of eukaryotic cells is a widely exploited process in research and biotechnology. Its molecular basis, however, remains incompletely understood. It is believed to rely on cellular DNA repair mechanisms, as it is favored by the presence of free DNA ends in the vector resembling double stranded breaks (DSBs). The two major pathways responsible for DSB repair in eukaryotic cells are non‐homologous end‐joining (NHEJ) and homologous recombination (HR) (Jackson, 2002). NHEJ is a fast mechanism that efficiently joins DNA ends with little processing (Mao et al., 2008). In contrast, HR is a slow, multi‐step process requiring resection of one of the two DNA strands and pairing to a homologous DNA template for repair. A third group of DSB repair pathways, believed to function when the main repair mechanisms are impaired, are collectively termed microhomology‐mediated end joining (MMEJ). MMEJ is a still poorly characterized family of pathways, also referred to as alternative or backup non‐homologous end‐joining (alt‐ or B‐NHEJ), which requires short (2–25 nt) homologies to align broken DNA strands before joining (Boboila et al., 2010; Gigi et al., 2014; Oh et al., 2014; Paul et al., 2013). Another hallmark of this process is the occurrence of large deletions and, less frequently, insertions of sequences copied from other parts of the genome, termed templated inserts (Ma et al., 2003; Merrihew et al., 1996). MMEJ shares DNA strand resection with HR, implying that it may partially rely on HR enzymes (Decottignies, 2007; Dinkelmann et al., 2009; Ma et al., 2003; Truong et al., 2013). Several mechanisms proposed to mediate chromosomal rearrangements associated with human genetic disorders were shown to rely on MMEJ (Costantino et al., 2014; Hastings et al., 2009; Hicks et al., 2010; Lee et al., 2007; Villarreal et al., 2012). Finally, another variant of MMEJ, termed synthesis‐dependent MMEJ (SD‐MMEJ), was also proposed to repair DSBs in the absence of pre‐existing homology (Yu and McVey, 2010). In this latter mechanism, the microhomologies required for the MMEJ pathway are synthetized de novo by an accurate non‐processive DNA polymerase. While all of these mechanisms may be mechanistically different, they possess several common features, such as the annealing of single stranded DNA ends at microhomology regions and the priming of low‐processivity DNA polymerization.

Plasmid integration into the genome of eukaryotic cells is an overall inefficient process, occurring in a minor proportion of cells that take up the exogenous DNA. It was shown to involve two major steps: (i) recombination between vector molecules to form multimeric transgene arrays termed concatemers and (ii) the recombination of the resulting concatemers with the genome, usually at a single or at few chromosomal loci (Folger et al., 1982; Grandjean et al., 2011; Kohli et al., 1998). The DSB repair pathways responsible for transgene concatemerization remain currently unclear. In mammalian cells, this process was attributed to HR (Folger et al., 1982; Wong and Capecchi, 1987), while NHEJ appeared to be involved in zebrafish embryos and rice (Dai et al., 2010; Kohli et al., 1998). In addition, some studies suggested that alternative pathways may also play a role in the joining of extrachromosomal DNA ends (Lundberg et al., 2001). Similarly, the mechanism mediating the recombination of the transgene with the genome remains to be fully identified. NHEJ is considered to mediate the majority of integration events in eukaryotic cells, while HR may be responsible for a smaller proportion of genomic integrations (Würtele et al., 2003). However, there is evidence that distinct repair pathways may also be implicated in this process (Iiizumi et al., 2008; Merrihew et al., 1996).

We previously reported that plasmid integration is enhanced by the presence of matrix attachment regions (MARs), which are epigenetic regulatory DNA elements that participate in the formation of chromatin boundaries and augment transcription (Galbete et al., 2009; Girod et al., 2007; Grandjean et al., 2011; Majocchi et al., 2014). MARs are thus widely used to sustain elevated transgene expression, as well as to prevent epigenetic silencing effects by blocking the propagation of heterochromatin (Allen et al., 2000; Harraghy et al., 2008; Zahn‐Zabal et al., 2001). Their action to increase genomic integration and plasmid copy number suggested that stimulating recombination may constitute an additional mechanism by which MARs increase transgene expression (Girod et al., 2007; Grandjean et al., 2011). Thus, in the present study, we sought to identify the pathway(s) responsible for the integration of MAR‐containing or ‐devoid plasmids into the genome of cultured cells.

Using siRNA‐mediated knock‐down approach, we show that a subset of alternative repair mechanisms resembling SD‐MMEJ may be preferentially used by CHO cells for the spontaneous integration of foreign DNA into their genome. This finding was confirmed by the characterization of plasmid‐to‐genome junction sequences, which were found to display an SD‐MMEJ pattern. Finally, we demonstrate that MAR elements and SD‐MMEJ favor transgene integration into permissive chromatin loci, and that the inhibition of competing recombination pathways can be used to improve the expression of recombinant proteins.

## Materials and Methods

### Cells, Plasmids, and siRNA

Adherent Chinese hamster ovary (CHO) DG44 cells (Urlaub and Chasin, 1980) were cultivated in DMEM/F‐12 + GlutaMAX™ supplemented with 1× HT and 10% fetal bovine serum (Gibco, Invitrogen), and with the antibiotic‐antimycotic solution (Sigma–Aldrich, #A5955). Suspension‐adapted CHO K1 derived cells (CHO‐M) were cultured in SFM4CHO (HyClone™) medium supplemented with 8 mM l‐Glutamine (PAA Laboratories GmbH) and 1× HT (Gibco).

The MAR‐devoid pGEGFP, MAR 1‐68‐containing p1‐68‐GFP, pGL3‐CMV‐DsRed, and pSVpuro expression vectors were described previously (Supplementary Fig. S1) (Grandjean et al., 2011). The HR and NHEJ reporter plasmids were kindly provided by V. Gorbunova (University of Rochester, New York) (Mao et al., 2008). The MMEJ‐specific GFP reporter assay, based on the pGEGFP vector, was constructed as described previously (Kostyrko and Mermod, 2015). Small interfering RNA duplexes, specifically designed to target the CHO cell homologs of the DNA repair proteins listed in Tables SI and SII, were designed and provided by Microsynth AG (Balgach, Switzerland) (Supplementary Table SIII). Three RNA duplexes were designed per mRNA to increase the probability of successful knock‐down. It was confirmed experimentally that individual siRNAs had similar effects on mRNA levels as the siRNA mixes, and it was also controlled that the siRNA and plasmids were delivered to the cells with above 90% efficiency by using a fluorescently labelled siRNA and a GFP expression plasmid (data not shown). Three negative (non‐targeting) siRNAs were designed as controls.

### Recombination Assays

For HR and NHEJ recombination transient assays, adherent CHO cells were transfected with HR or NHEJ reporter plasmids digested with I‐SceI, and with the pGL3‐CMV‐dsRed plasmid to normalize for transfection efficiency, using Fugene 6 (Promega). The pGEGFP plasmid was transfected in parallel as a positive control of GFP expression.

For siRNA‐mediated knock‐downs of DNA repair proteins, adherent CHO DG44 cells were transfected with equimolar mixes of three mRNA‐specific or control siRNA duplexes at a final concentration of 50 nM using Lipofectamine RNAiMAX (Invitrogen), according to manufacturer's instructions (Supplementary Fig. S2A). After 2 days, the siRNA‐treated cells were re‐transfected with pGEGFP or p1‐68‐GFP, and with a puromycin resistance plasmid pSVpuro (Clontech), using Lipofectamine 2000 (Invitrogen). Prior to transfection all plasmids were linearized with PvuI and purified by ethanol precipitation. Puromycin (5 μg/mL) was added to the culture medium 24 h after transfection, and stably transfected cells were selected for 2 weeks. Stable GFP expression was analyzed by flow cytometry (CyAn flow cytometer, Beckman Coulter), whereas aliquots of each sample were used for genomic DNA extraction.

### Colony Formation Assay

To assess the frequency of genomic integration events, CHO DG44 cells were transfected with siRNA duplexes against selected DNA repair proteins, using the protocol described above (Supplementary Fig. S2A). Cells were re‐transfected 72 h later with pGEGFP or p1‐68‐GFP, and pSVpuro, using Lipofectamine 2000 (Invitrogen), once the effects of the knock‐down on cell cycle progression had disappeared (Kostyrko et al., 2015). The cells were trypsinized and counted 24 h after the second transfection, and 10000 viable cells were seeded in complete medium into each well of a 6‐well plate. Puromycin (5 μg/mL) was added to the medium 7 h after seeding. After 10 days of selection, puromycin‐resistant colonies were stained with 0.2% methylene blue and quantified using ImageJ (U.S. National Institutes of Health, Bethesda, MD).

### Transgene Copy Number Determination and Quantitative PCR

To analyze the transgene copy number, total genomic DNA was isolated from cells using the DNeasy purification kit (Qiagen). For quantitative PCR (qPCR), 6 ng of genomic DNA were analyzed using the SYBR Green I Master kit for the Light Cycler 480 machine (Roche) using AGCAAAGACCCCAACGAGAA and GGCGGCGGTCACGAA as GFP‐specific primers. The beta‐2‐microglobulin (B2M) CHO gene was amplified as a normalization control using ACCACTCTGAAGGAGCCCA and GGAAGCTCTATCTGTGTCAA as primers. The number of integrated transgene (GFP) copies was calculated using the B2M gene as a reference, as previously described (Pfaffl, 2001).

### Characterization of Transgene Integration Sites

To assess which CHO genes were expressed in our culture conditions, the transcriptome of the suspension‐adapted parental CHO K1 cells was determined by paired‐end sequencing using the Illumina technology by the Next Generation Sequencing Facility of the University of Lausanne. Expressed coding sequences were annotated using the Annotation Release 101 of the Chinese hamster genome assembly (CriGri_1.0, GCF_000223135.1) (Xu et al., 2011).

To identify the plasmid integration sites in polyclonal populations, CHO K1 cells were electroporated with the MAR‐devoid pGEGFP or the MAR‐containing p1‐68‐GFP plasmids and with the pSVpuro puromycin resistance construct using the Neon® transfection system (Invitrogen). After 3 weeks of puromycin selection, total genomic DNA was isolated from polyclonal cells using the Genomic‐tip G/20 kit (Qiagen). The DNA was sequenced using the Single Molecule Real‐Time (SMRT) technology (Pacific Biosciences) at the Next Generation Sequencing Facility of the University of Lausanne. CHO cells transfected with p1‐68‐GFP were sequenced using 20 SMRT cells, and those transfected with pGEGFP required the use of 60 SMRT cells to obtain a similar number of integration site sequences. Transgene integration sites were identified by a custom identification pipeline. PacBio filtered subreads were obtained using the tool DEXTRACTOR (Myers, unpublished) using the standard settings. Plasmid sequences were identified in PacBio filtered subreads with the help of the alignment tool BLASR (Chaisson and Tesler, 2012). A raw score of at least −500 was chosen as cut‐off based on results using PacBio reads from untransfected CHO cells. Flanking regions of matching plasmid sequences were extracted and mapped onto the CHO K1 genome using BLASR. 14 CHO genomic integration sites were identified in the p1‐68‐GFP‐transfected population and 10 in the pGEGFP‐transfected population. Two sets, one of 14 and one of 10, different, randomly picked genomic scaffolds of the same length (±10%) as the sample scaffolds were selected as controls. The Annotation Release 101 of the Chinese hamster genome assembly (CriGri_1.0, GCF_000223135.1) was used to identify the CHO genes in the vicinity of the integration sites. The presence of genes near the plasmid integration position in each of the identified scaffolds was compared with an analogous position on a corresponding control scaffold. An exact binomial test was used to calculate statistical significance between these datasets. Based on this analysis, integration within 5 kb from an open reading frame (ORF) was considered as intragenic, whereas integration within 35 kb from an ORF was defined as gene‐proximal.

Suspension‐adapted CHO K1 cells were stably transfected in multiple transfection cycles with plasmid vectors containing the human MAR X‐29 and encoding the light and heavy chains of the trastuzumab and adalinumab therapeutic antibodies, as previously described (Le Fourn et al., 2014), with prior PvuI cleavage of the vectors. Clones expressing the highest amount of the recombinant proteins were selected for whole genome sequencing (Illumina), performed by Fasteris SA (Plan‐Les‐Ouates, Switzerland). Integration sites were first predicted by the in silico identification of paired reads displaying linked plasmid and genomic sequences, and the predicted junctions were subsequently validated by PCR amplification and Sanger sequencing. Identification of CHO genes near the plasmid integration sites was performed as described for the polyclonal populations.

### Analysis of Immunoglobulin‐Expressing CHO Cells

To assess the impact of DNA repair protein knock‐down on recombinant protein expression, CHO K1 cells were electroporated with a negative control siRNA and siRNAs against MDC1, Ligase I, Rad51, and Rad52 using the Neon® transfection system (Invitrogen) (Supplementary Fig. S2B). Two days post transfection the cells were electroporated with PvuI‐linearized human immunoglobulin (IgG1) expression vectors containing the MAR 1–68 and a puromycin resistance plasmid (pSVpuro) (Supplementary Fig. S1), using the Neon® transfection system (Invitrogen). After 3 weeks of antibiotic selection the IgG titer in cell culture supernatants was measured by sandwich ELISA and the specific productivity was calculated as described previously (Le Fourn et al., 2014).

## Results

### Plasmid Integration Does Not Rely on NHEJ or the Canonical HR Pathway

To assess the possible implication of NHEJ and HR in plasmid concatemer formation and spontaneous integration into the cell genome, we silenced the components of these major DSB repair pathways in CHO DG44 cells using short interfering RNA (siRNA) (Supplementary Fig. S3 and Table SI). Efficient reduction of the target mRNA and/or protein levels by siRNA transfection was validated experimentally, to insure decreased levels by at least twofold (Supplementary Figs. S4 and S5).

To evaluate if the knock‐down of these genes affects DNA recombination, we used previously described HR and NHEJ fluorescent reporter assays based on the repair of transiently transfected plasmids with a I‐SceI‐induced DSB in the GFP coding sequence (Mao et al., 2008; Seluanov et al., 2004). These assays enable to evaluate the efficiency of extrachromosomal break repair, and thereby may provide an estimation of HR and NHEJ involvement in plasmid concatemer formation. We observed that DSB repair of the HR reporter plasmid was impaired by the knock‐down of the Rad51 HR protein, whereas it was rather increased in cells treated with siRNAs targeting NHEJ factors (Supplementary Fig. S6A). This indicated that Rad51 may contribute to the repair of DSBs in episomal plasmids. Interestingly, the knock‐down of the remaining HR factors had no detectable effect on GFP reconstitution in this assay, although there was a very significant difference between the overall effect of knocking‐down NHEJ and HR genes, in line with the previously reported competition between these pathways (Neal et al., 2011). In contrast, the occurrence of GFP expression from the NHEJ reporter was not altered by any of the NHEJ‐targeting siRNAs (Supplementary Fig. S6B), implying that NHEJ is not prominently used to rejoin episomal DSBs in CHO cells or that alternative end‐joining pathways may be more active than NHEJ.

We further assessed the recombination mechanisms involved in plasmid concatemer formation and genomic integration by stably transfecting the siRNA‐treated CHO cells with plasmids carrying the GFP reporter and a puromycin resistance gene (Supplementary Fig. S2A). The average number of integrated GFP copies was measured in antibiotic‐resistant polyclonal populations, so as to assess the efficiency of plasmid concatemerization prior to genomic integration. Since expression from individual plasmids can be influenced by the surrounding chromatin environment, the level of GFP fluorescence and its normalization to the transgene copy number was used to estimate plasmid integration within transcription permissive or non‐permissive areas of the genome. Finally, we measured the efficiency of plasmid genomic integration by quantifying puromycin‐resistant colonies arising from cells that had successfully integrated transgenes into their genome, focusing on siRNAs that affected GFP expression or plasmid concatemerization, as well as representative targets from each DSB repair pathway.

**Figure 1:**
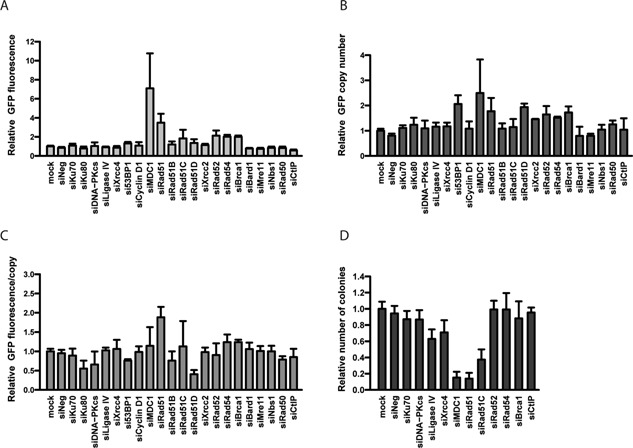
Effect of HR and NHEJ components knock‐down on plasmid genomic integration and expression. CHO cells treated with indicated siRNAs were re‐transfected with a GFP expression plasmid and puromycin resistance vector. Puromycin‐resistant stable polyclonal CHO populations were assessed for average GFP fluorescence (**A**), GFP copy number (**B**), GFP expression per transgene copy (**C**), and the occurrence of puromycin‐resistant colonies (**D**). Values represent mean fold change over control cells not treated with siRNAs (mock); s.e.m error bars, *n* ≥ 3.

The average GFP expression and plasmid copy number were not affected by the down regulation of NHEJ activities such as DNA‐PKcs, Ligase IV or Xrcc4, nor was the expression per transgene copy or the number of antibiotic resistant colonies (Fig. 1A–D). This indicated that NHEJ activities are not limiting for plasmid concatemerization and integration within the cell genome.

Stable GFP expression and/or transgene copy numbers were increased by the knock‐down of HR proteins, notably MDC1, Rad51, Rad52, Rad54, and Brca1 (Fig. 1A and B). The knockdown of these proteins had overall little effect on gene expression when normalized to the copy number, indicating that the increased expression observed upon HR gene knockdown resulted mostly from an increased copy number rather than from preferential plasmid integration into transcription‐permissive chromatin (Fig. 1C). These observations indicated that HR activities may oppose a mechanism that mediates plasmid concatemerization prior to genomic integration. However, the knock‐down of proteins having an effect on plasmid concatemerization and/or GFP expression, such as MDC1 and Rad51, strongly decreased the number of puromycin‐resistant colonies (Fig. 1D), indicating that these components of the HR pathway may mediate transgene genomic integration. Interestingly, the frequency of integration was not affected by the knock‐down of other components of HR, such as Rad52, Rad54, or Brca1, despite their effect on transgene concatemerization and expression. These findings implied that some HR activities are required for genomic integration whereas others are not, suggesting the occurrence of non‐canonical HR‐related integration mechanisms.

### MMEJ‐Type Mechanisms Mediate Plasmid Concatemerization and Genomic Integration

Given that neither the NHEJ nor the canonical HR pathway may be involved in plasmid concatemerization prior to genomic integration, we speculated that this could involve MMEJ‐related mechanisms active in eukaryotic cells with impaired NHEJ and/or HR, but that may share early 5′ strand resection events with the HR pathway (Supplementary Fig. S3 and Table SII) (Decottignies, 2007; Ma et al., 2003).

**Figure 2:**
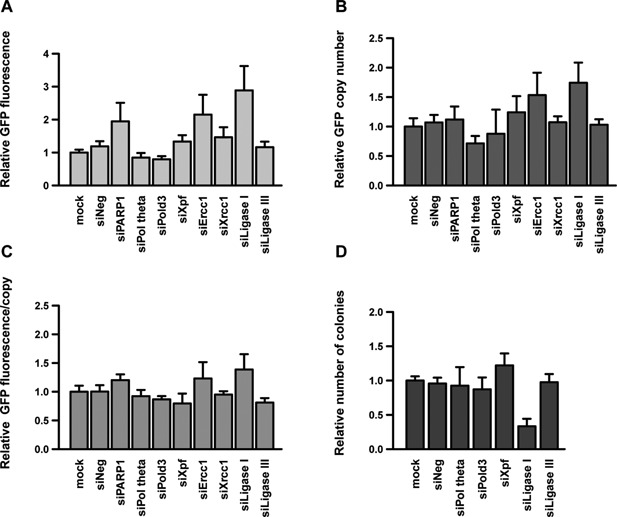
Effect of MMEJ components knock‐down on plasmid genomic integration and expression. CHO cells were treated with siRNAs against the indicated MMEJ genes and processed as described in the legend to Figure 1. The average GFP fluorescence (**A**), GFP copy number (**B**), GFP expression per transgene copy (**C**), and frequency of genomic integration events (**D**) were assessed and represented as in Figure 1 (*n* ≥ 3).

Knock‐down of most MMEJ proteins had a moderate effect on plasmid integration or expression, possibly because these pathways may be masked by other repair mechanisms in the absence of induced DNA damage, as was the case here (Fig. 2A–C). Nevertheless, we observed a small decrease in GFP copy number upon the knock‐down of DNA polymerase θ (Pol theta), suggesting that this polymerase might be involved in plasmid concatemerization, although the potential involvement of other DNA polymerases cannot be excluded. Interestingly, the knock‐down of Ligase I had an opposite effect (Fig. 2A and B). Moreover, the depletion of this ligase strongly inhibited plasmid genomic integration (Fig. 2D). A recent study suggested the existence of two branches of the MMEJ‐related end‐joining pathways, one of which may depend on Ligase I whereas the other would require Ligase III (Oh et al., 2014; Paul et al., 2013). We thus speculated that upon Ligase I knock‐down, the Ligase III‐dependent branch could prevail, which may favor plasmid concatemer formation. In contrast, the pathway responsible for plasmid genomic integration may be dependent on Ligase I, as it is suppressed by it's depletion.

We have recently constructed a MMEJ‐specific GFP reporter assay, based on principles analogous to the HR and NHEJ reporter plasmids used above (Kostyrko and Mermod, 2015). Interestingly, the use of this reporter in CHO cells revealed that the majority of episomal DSBs were not re‐joined by a simple MMEJ pathway. Instead, the joined sequences of most repaired vectors rescued from the transfected cells resembled the recently proposed alternative DNA synthesis dependent (SD)‐MMEJ mechanism (Yu and McVey, 2010). This pathway relies on a non‐processive DNA polymerase, such DNA polymerase θ, to copy short homologous sequences (2–9 bp) from a different part of the repaired molecule, which can then be used to rejoin the DSB (Yousefzadeh et al., 2014; Yu and McVey, 2010). As a result, the junction sequence consists of a short duplication (direct or inverted) of a sequence found nearby on the repaired DNA fragment (Supplementary Fig. S3). Seventy percent of the analyzed repair products had no pre‐existing microhomology indicative of MMEJ, but they displayed direct or inverted repeat sequences associated SD‐MMEJ, up‐ or downstream of the repaired junction (Kostyrko and Mermod, 2015). We thus concluded that plasmid‐to‐plasmid joining relies mostly on a SD‐MMEJ pathway potentially involving DNA polymerase θ and Ligase III, and that the simple MMEJ mechanism is seldom used.

### MAR Elements Promote Plasmid Integration by Stimulating SD‐MMEJ Pathways

We previously showed that transgene integration in CHO cells is enhanced three‐ to fourfold in the presence of matrix attachment regions (MARs), which are DNA elements that form chromatin domain boundaries (Girod et al., 2007; Majocchi et al., 2014). A human MAR, termed MAR 1–68, was found to increase both the number of transgene copies as well as the frequency of genomic integration events in CHO cells, which has been previously ascribed to HR‐related mechanisms (Grandjean et al., 2011). However, which HR‐related recombination mechanism may be activated by MAR elements was not assessed.

**Figure 3:**
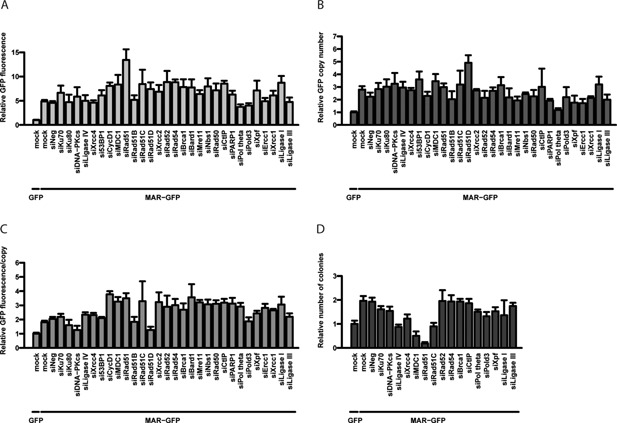
Effect of a MAR element and recombination gene knock‐down on plasmid genomic integration and expression. The effect of the inclusion of a MAR element on stable GFP expression (**A**), GFP copy number (**B**), GFP expression per transgene copy (**C**), and the frequency of genomic integration events (**D**), were assessed as described for Figures 1 and 2, except that siRNA‐treated cells were re‐transfected with GFP or MAR‐GFP vectors, as indicated (*n* ≥ 3).

To unambiguously identify the recombination mechanism activated by such elements, we combined the addition of the human MAR 1–68 in the GFP vector with the siRNA knock‐down approach used earlier. As shown previously, inclusion of the MAR 1–68 enhanced GFP expression and copy number by approximately five‐ and threefold, respectively, when compared to the MAR‐devoid control (Fig. 3A and B). This indicated that the MAR acted in part to activate plasmid concatemerization, whereas it concomitantly increased expression per gene copy (Fig. 3C). The presence of the MAR also increased by around twofold the proportion of cells having recombined the transgenes into their genome (Fig. 3D), indicating that it also activated genomic integration.

In the presence of the MAR, the silencing of NHEJ factors had no effect on transgene expression or copy number, as before (Fig. 3A and B). In contrast, the knock‐down of many HR and cell cycle control factors yielded very high transgene expression, but without further increasing the transgene copy number. Consistently, we observed an enhancement of expression per gene copy upon the knock‐down of most HR factors, which was markedly higher than the increase already mediated by the MAR (Fig. 3C). A strong inhibition of the frequency of plasmid genomic integration was again noted upon the knock‐down of MDC1 and especially Rad51 (Fig. 3D). This indicated that these factors and the MAR may act synergistically to promote transgene genomic integration. However, upon the knock down of Rad51 and other HR proteins, the MAR‐containing plasmids may have integrated preferentially into expression‐permissive portions of the genome. We therefore speculated that the MAR acts to promote one or several MMEJ‐related pathways that may direct transgenes into expression‐favoring chromatin structures.

In the presence of the MAR, the knock‐down of MMEJ factors had mostly similar effects on GFP expression and copy number as observed earlier for the MAR‐devoid plasmid, with a small decrease upon the knock‐down of DNA polymerase θ, and an increase in the absence of Ligase I (Fig. 3A and B). Interestingly, the presence of the MAR seemed to counteract the effect of Ligase I down‐regulation on transgene genomic integration, possibly by reducing the inhibitory effect of the reduced ligase level, or by stimulating a distinct recombination mechanism (Figs. 2C and 3D). Overall, we concluded that the MAR may activate both concatemerization and genomic integration processes by stimulating SD‐MMEJ‐related repair pathways, and that these pathways may concur with the MAR to favor integration into expression‐permissive genomic loci.

### The MAR and SD‐MMEJ Pathways Mediate Transgene Integration Near Cellular Genes

To further assess which of the alternative recombination pathways may mediate favorable genomic integration events, we analyzed the genomic integration loci and the DNA sequence of the genome‐plasmid junctions. This was performed on three CHO clones transfected multiple times with immunoglobulin (IgG) expression vectors containing the human MAR X‐29 and selected for high stable expression of the therapeutic protein. To do so, we used a whole genome sequencing approach on these clones and devised a software to identify paired sequence reads pertaining to the plasmid and the CHO genome. Six integration sites in one clone (BS01) and two in the other clones (BS03 and Cp33/64) were predicted in silico and validated experimentally by PCR amplification and DNA sequencing. The occurrence of the predicted number of plasmid integration loci was further validated by FISH for two of the analyzed clones (Supplementary Fig. S7).

**Figure 4:**
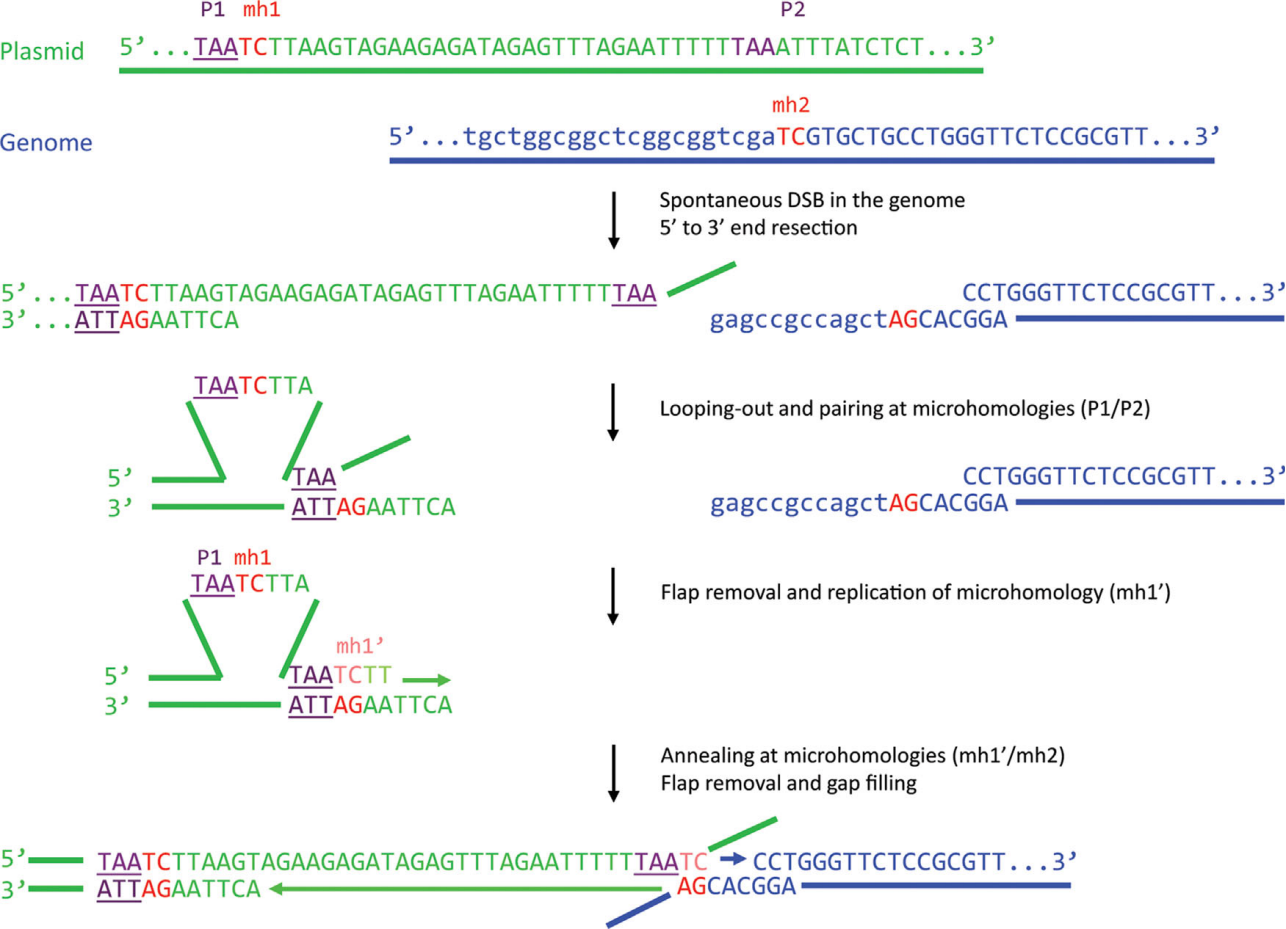
Example of a plasmid‐to‐genome junction and underlying SD‐MMEJ mechanism. The integration site and junction sequence used in this example is taken from Supplementary Table SIV (clone BS01, integration site #2, right junction). P1/P2, primer repeats; mh1/mh2, microhomology repeats. Adapted from Yu and McVey (2010).

**Figure 5:**
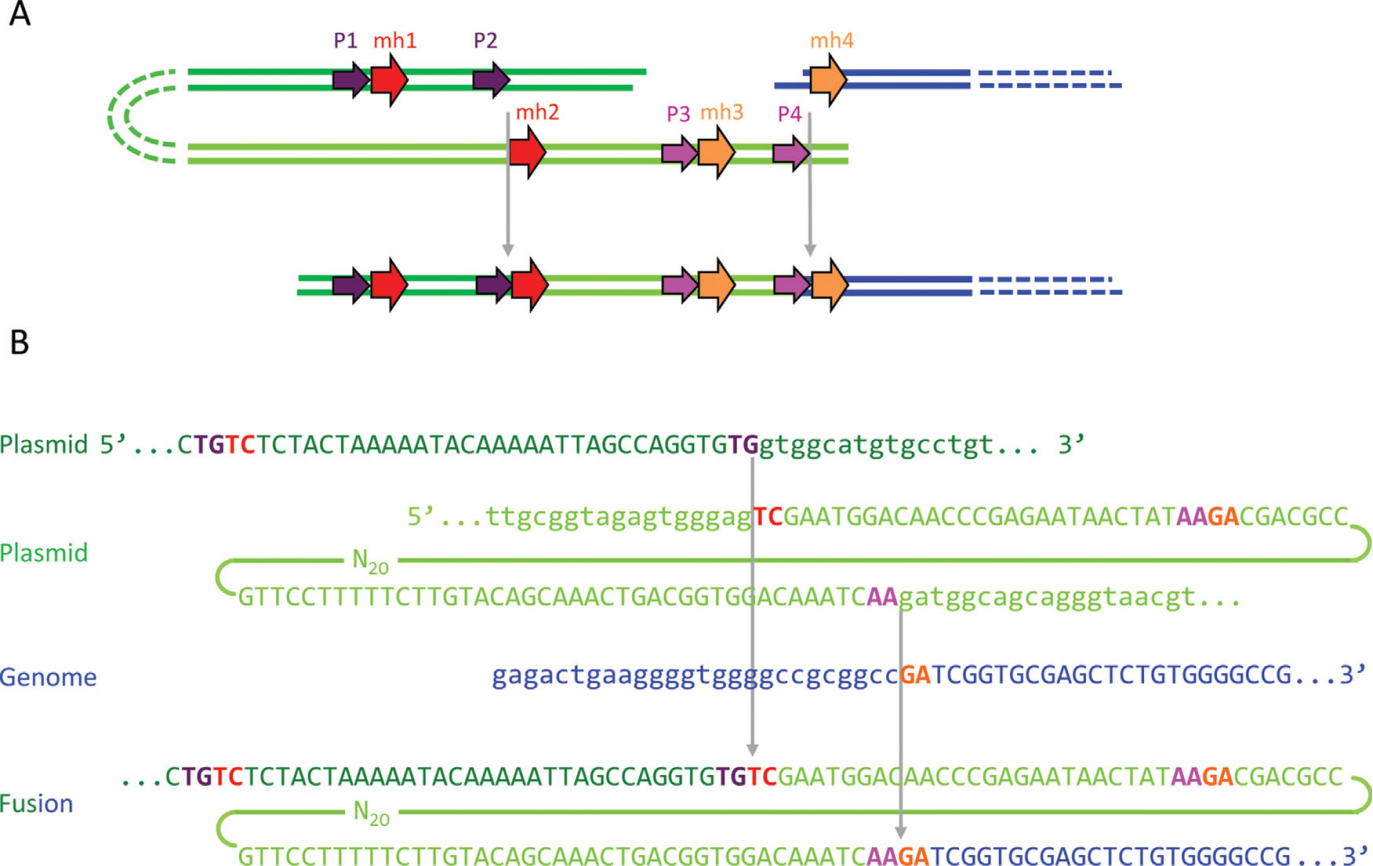
Example of a plasmid‐to‐genome junction and SD‐MMEJ mechanism requiring a templated insertion. (**A**) A scheme showing the mechanism of plasmid (dark green) joining with the genome (blue). Another fragment of the plasmid (light green) serves as an adaptor providing the microhomologies required for joining and becomes incorporated into the junction as a templated insert. (**B**) Sequences of plasmid and genome fragments shown in panel A. The integration site and junction sequence used in this example is taken from Supplementary Table SIV (clone BS01, integration site #1, right junction). P1/P2 and P3/P4, primer repeats; mh1/mh2 and mh3/mh4, microhomology repeats.

From the five integration sites where the junction sequences were validated experimentally on both sides of the transgenes, two had large deletions (913 bp in BS01 and 320 bp in Cp33/64), as expected from MMEJ‐related mechanisms (Supplementary Table SIV and Fig. 4). In 5 of the 15 experimentally validated junctions, we noted the presence of short (1–3 bp) or long (60–100 bp) templated inserts, suggesting the involvement of a DNA polymerase in the repair process, a hallmark of the SD‐MMEJ mechanism (Fig. 5). All analyzed junction sequences fitted well to the SD‐MMEJ model, although 5 out of 15 junctions also covered pre‐existing microhomologies (≥2 nt), and thus could also be explained by simple MMEJ. Interestingly, no integration site could be explained by HR. Although NHEJ cannot be fully excluded, as it does not strictly require extensive homology, the SD‐MMEJ mechanism more readily explains the presence of extended deletions and templated inserts. Moreover, no junction lacking any type of microhomology was observed. Overall, these results confirmed that the genomic integration of MAR‐containing plasmids predominantly involves a SD‐MMEJ pathway.

Out of the 10 integration events, eight had occurred within or near cellular genes, whereas only two were intergenic. Seven out of these eight gene‐proximal integrations were found in or close to an expressed gene (Supplementary Table SV), suggesting that most integration events had occurred in transcriptionally active genomic loci. These results further suggested that the MAR‐containing plasmids preferably integrate within‐ or in close proximity‐ to expressed CHO genes. To assess whether this indeed resulted from the presence of the MAR in the IgG expression vector or from the selection of highly expressing clones, we directly compared the integration loci of MAR‐containing and MAR‐devoid GFP expression vectors in polyclonal cell populations.

Analysis of the integration sites identified from the whole genome sequencing of these cells revealed that, in presence of the MAR, plasmids indeed often integrated close to cellular genes (10/14 loci) (Supplementary Table SV and Fig. S8). This result was significantly different from random (*P* = 0.05), indicating that the MAR may stimulate genomic integration into chromatin regions permissive for transgene expression. In the cells transfected with the MAR‐devoid plasmid, integration in the vicinity of genes was not significantly enriched (Supplementary Table SV and Fig. S8B). Furthermore, these cells required threefold more sequencing reads to identify a comparable number of integration loci as obtained from the cells transfected with the MAR, further indicating that genomic integration events were less frequent in the absence of the MAR.

Interestingly, all cellular genes near the integration loci of MAR‐devoid plasmids were transcribed in the parental CHO cells (Supplementary Table SV and Fig. S9). This suggested that, in the absence of the MAR, the cells had to integrate the transgenes into transcriptionally active chromatin in order to express the selection gene at a sufficient level to survive antibiotic selection. This may explain the strong decrease in cell survival upon the knock‐down of Rad51, as this protein was recently reported to be primarily responsible for DSB repair in transcriptionally active chromatin (Aymard et al., 2014). In contrast, presence of the MAR seemed to alleviate the need to integrate transgenes into transcribed genomic sequences, as only half of the CHO genes close to integration sites were found to be transcriptionally active. This indicated that the MAR itself may ensure high expression of transgenes integrated in non‐transcribed DNA, likely due to its previously reported transcription‐enhancing properties (Galbete et al., 2009; Majocchi et al., 2014). Taken together, these results suggested that MAR elements may promote transgene integration into gene‐rich chromatin regions by stimulating an SD‐MMEJ mechanism.

### MARs and HR or SD‐MMEJ Knock‐Down Improve Recombinant Protein Expression

**Figure 6:**
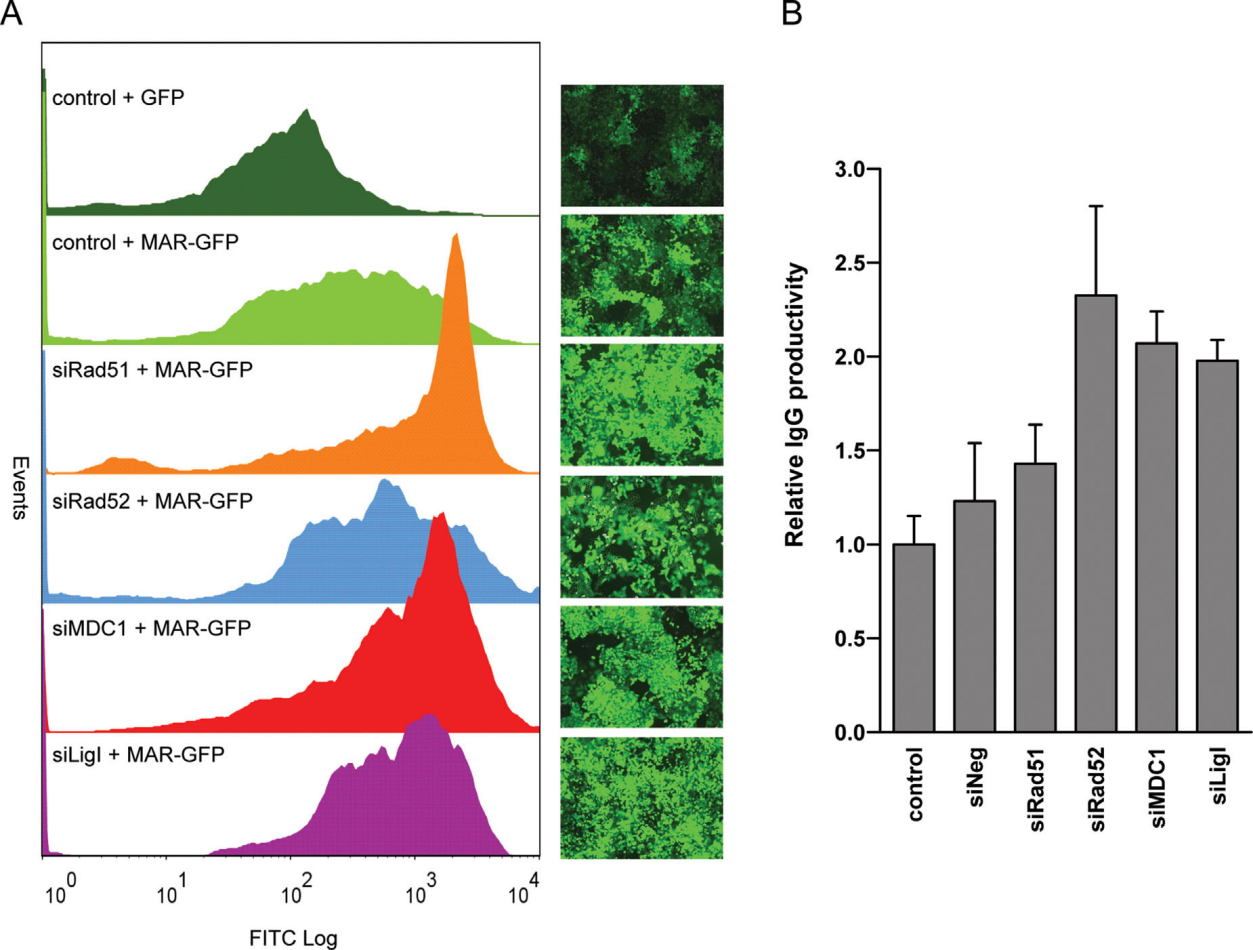
Engineering of the transgene integration process for improved expression. (**A**) Adherent CHO cells transfected with the indicated siRNAs or left untreated (control), were re‐transfected with a GFP or MAR‐GFP vector, as indicated, and selected for antibiotic resistance. GFP fluorescence profiles of polyclonal cell pools and corresponding fluorescence microscopy pictures are shown. (**B**) Specific IgG productivity in polyclonal, suspension‐adapted CHO cells treated as for panel A, except that they were re‐transfected with the MAR‐containing IgG1 expression vectors. Values represent the average fold change in IgG secretion (in picograms/cell/day) as compared to the cells not treated with siRNAs (control); s.e.m error bars, *n* = 3.

The transient knock‐down of MDC1, Rad51, Rad52, and Ligase I was found to mediate the highest and most homogeneous GFP fluorescence from polyclonal pools of cells stably transfected with the MAR‐GFP vector (Fig. 6A). To ascertain whether the knock‐down of these specific HR and/or SD‐MMEJ activities may be used in conjunction with MAR elements as a general approach to boost the expression of recombinant proteins, we similarly assessed vectors encoding a therapeutic IgG1 immunoglobulin, using a suspension‐adapted CHO K1 cell line derivative suitable for the production of therapeutics. CHO cells treated with siRNAs against Rad51, Rad52, MDC1, or Ligase I were subsequently re‐transfected with MAR‐containing vectors for the human IgG1 light and heavy chains (Supplementary Fig. S2B). Polyclonal populations were then assessed for specific antibody secretion, which revealed that prior treatment with Rad52, MDC1, or Ligase I siRNAs increased stable IgG expression by approximately twofold relative to the untreated cells (Fig. 6B). The high productivity levels observed from these polyclonal populations, up to over six picograms per cell per day (PCD), are usually only observed from monoclonal populations obtained from the screening of hundreds of individual cell clones, to identify the most productive ones. Interestingly, Rad51 depletion in CHO K1 cells had a weaker effect on transgene expression than in CHO DG44 cells. This could be due to the combination of mechanical stress associated with growth as cell suspension in shake flasks, antibiotic selection and the deleterious effect of Rad51 knock‐down. Consistently, we observed that CHO K1 cells treated with Rad51 siRNA grew much slower than the cells treated with other siRNAs, and only a small number of cells survived selection (data not shown). We hypothesize that the population of CHO K1 cells that recovered from selection represented cells which retained some Rad51 activity, and which thus did not have a large increase of plasmid concatemerization and overall expression.

In conclusion, the increase in expression mediated by Rad52, MDC1, and Ligase I knock‐down could be observed for distinct recombinant proteins, and from the use of distinct CHO cell lines and vectors. We concluded that the production of therapeutic proteins in CHO cells may be significantly improved by transiently altering their DSB repair properties during transfection and by incorporating MAR elements in the vector.

## Discussion

Eukaryotic cells have developed many defense mechanisms that detect and repair DNA double stranded breaks, one of the most deleterious types of DNA damage. The two canonical pathways responsible for DSB repair are HR and NHEJ. However, recent evidence indicated that these two mechanisms may not suffice to repair all DSBs, and that several alternative pathways, collectively termed MMEJ or alt‐NHEJ, also exist in eukaryotic cells (Gigi et al., 2014; Truong et al., 2013). These later processes are often obscured by the main repair mechanisms, which may predominate in normal cells. Furthermore, their components are still poorly characterized and there was no simple assay to specifically detect them, rendering their study difficult (Kostyrko and Mermod, 2015). However, they are now attracting increasing attention, notably in oncology, since these “illegitimate” recombination pathways were shown to be more prevalent in tumor cells and to cause chromosomal rearrangements leading to cancer (Bentley et al., 2004; Simsek et al., 2011; Tobin et al., 2012; Zhang and Jasin, 2011).

Here, we found that NHEJ and HR are not the main pathways responsible for non‐specific recombination in CHO cells, as required for plasmid genomic integration in these cells. Rather, we found that the absence of several HR factors augmented plasmid concatemerization, implying that HR proteins may compete with one or more DSB repair pathways that mediate this process. In contrast, specific HR proteins, such as Rad51, were required for efficient transgene recombination with the genome, whereas the silencing of downstream HR proteins had no effect. This suggested the involvement of other mechanisms, distinct from the canonical NHEJ or HR pathways but nevertheless requiring DNA homology, such as MMEJ‐related pathways. Consistently, the knock‐down of Ligase I, a protein reported to play a role in alternative DSB repair pathways, was found to alter plasmid genomic integration.

**Figure 7:**
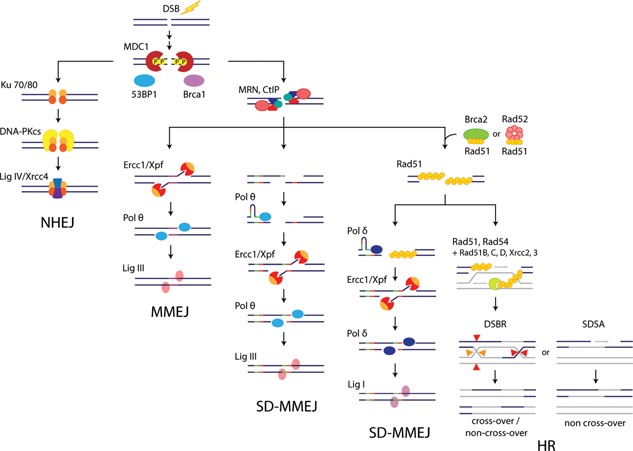
Revised model of the major CHO cell DSB repair pathways. A novel model describing the possible interplay of the NHEJ, HR, MMEJ, and two distinct SD‐MMEJ pathways involved in DSB repair in CHO cells, as modified from Supplementary Figure S3. Although the junction sequences resulting from both SD‐MMEJ pathways are similar, the Ligase I‐dependent SD‐MMEJ requires the homology‐searching Rad51 protein, and it may provide a fallback mechanism in the absence of extensive homology, as required to complete HR. Activities that initiate MMEJ and Ligase III‐dependent SD‐MMEJ remain to be identified, but the presence or absence of pre‐existing microhomologies may dictate the choice between these two pathways.

As the majority of rejoined plasmid extremities displayed microhomology patterns and templated inserts, we attribute these end‐joining events to the SD‐MMEJ mechanism proposed by Yu and McVey (2010). Indeed, both plasmid‐to‐plasmid and plasmid‐to‐genome fusion sequences were also present as direct or inverted repeats near the junctions, occasionally accompanied by templated inserts. However, the knock down of specific SD‐MMEJ activities had distinct effects on plasmid concatemer formation and on genomic integration, suggesting the occurrence of multiple SD‐MMEJ pathways. One of these pathways, which may rely on DNA polymerase θ and Ligase III, appears to mediate plasmid concatemerization. The other SD‐MMEJ pathway, which may involve the activity of Ligase I, appears to mediate the recombination of plasmid concatemer with the genome, as indicated by the finding that the lack of this ligase nearly abolished genomic integration of the GFP vector. Taken together, these results imply that concatemer formation and integration of MAR‐devoid plasmids may be mediated by sets of proteins belonging to distinct branches of the SD‐MMEJ pathways, as proposed in Figure 7.

Interestingly, we observed that the knock down of Rad51 had similar effects as the silencing of Ligase I, implying that they contribute to the same pathway mediating genomic integration of exogenous DNA. The mechanism mediating microhomology search of the SD‐MMEJ pathway remains mostly uncharacterized, but it may involve DSB repair components that are common to other mechanisms. In this model, the Ligase I‐dependent SD‐MMEJ pathway may lie downstream of the search for a homologous DNA strand by Rad51, as in canonical HR (Fig. 7). However, the lack of extended homology may preclude the productive cooperation of Rad51 with its accessory proteins, preventing extended strand invasion and the successful completion of HR. End‐joining would then rather be performed by Ligase I‐dependent SD‐MMEJ, as a salvage repair pathway, since it only requires short homology regions as shared by the plasmid and cell genome. When such microhomologies are not available, they may be provided by an adaptor DNA stretch copied from nearby plasmid or genome sequences, leading to the insertion of a templated insert separating the joined sequences (Fig. 5). We hypothesize that the enzyme involved in the synthesis of the templated insert may be DNA polymerase δ, which together with Ligase I participates in DNA replication and long patch base excision repair (BER) (Stucki et al., 1998). In human cells, a break‐induced replication (BIR) mechanism responsible for the repair of one‐ended DSBs was also recently shown to rely on POLD3, a DNA polymerase δ subunit, and to involve microhomologies (Costantino et al., 2014; Lydeard et al., 2007). Nevertheless, whether the Rad51‐dependent SD‐MMEJ pathway proposed here may be related to BIR remains to be established.

Inclusion of a MAR element also increased plasmid concatemerization, suggesting that it can act to activate the processing of linearized plasmid extremities by a Ligase I‐independent SD‐MMEJ mechanism. In addition, the MAR presence increased genomic integration and dampened the inhibitory effect of Ligase I downregulation, whereas it did not abolish the requirement for Rad51. These findings suggest a preferential use of the Ligase I‐dependent SD‐MMEJ mechanism for the genomic integration of the MAR‐devoid plasmid, whereas the presence of the MAR may stimulate the use of a repair pathway downstream of Rad51 that may involve a distinct ligase, for example, Ligase III.

The molecular mechanisms by which MARs may promote SD‐MMEJ‐mediated recombination could involve their AT‐rich cores, which possess a high potential for double helix denaturation (Bode et al., 1992; Platts et al., 2006), or their enrichment in topoisomerase II cleavage sites and so‐called fragile sites that may be the hot spots of DNA breakage and repair (Jackson et al., 2003; Sperry et al., 1989; Svetlova et al., 2001). Consistently, these sites were previously reported to be preferred targets of plasmid integration (Rassool et al., 1991). MAR elements were also proposed to mediate DNA replication initiation in mammalian somatic cells (Debatisse et al., 2004). Thus, they might associate with DNA replication machinery components also involved in MMEJ‐related mechanisms (e.g., Pold3, Ligase I), thereby contributing to the repair of DSBs arising at replication forks (Truong et al., 2013).

In this study, we identified SD‐MMEJ as the primary mechanism driving plasmid integration in the genome of CHO cells. We propose the occurrence of two distinct SD‐MMEJ branches relying on different subsets of proteins, both of which are stimulated by MAR elements, to increase transgene copy number and to preferentially target plasmid DNA into potentially expression‐permissive, gene‐rich regions of the genome. Finally, we use this knowledge to transiently modify the DNA recombination properties of CHO cells to improve the expression of a therapeutic antibody, demonstrating that this approach can be used to engineer cells for more efficient recombinant protein expression.

The authors wish to thank V. Gorbunova for the kind gift of plasmid reagents. We thank the Vital‐IT high performance computer cluster and the University of Lausanne Genomic Technologies Facility for expert support. This work was supported by a grant from the Swiss Government Commission for Technology and Innovation and Selexis SA (grant 13939.1 PFLS‐LS), and by the University of Lausanne.

## Supporting information

Additional supporting information may be found in the online version of this article at the publisher's web‐site.

Supplementary Figures

Supplementary Tables
