# Supplementary material for: MAR‐Mediated transgene integration into permissive chromatin and increased expression by recombination pathway engineering
Source: Biotechnol Bioeng. 2016 Oct 3;114(2):384–96. doi: 10.1002/bit.26086 (PMC5215416; doi:10.1002/bit.26086)
Supplement: Supplementary file 1 — Supplementary Figures [file BIT-114-384-s001.pptx]

## Slide 1
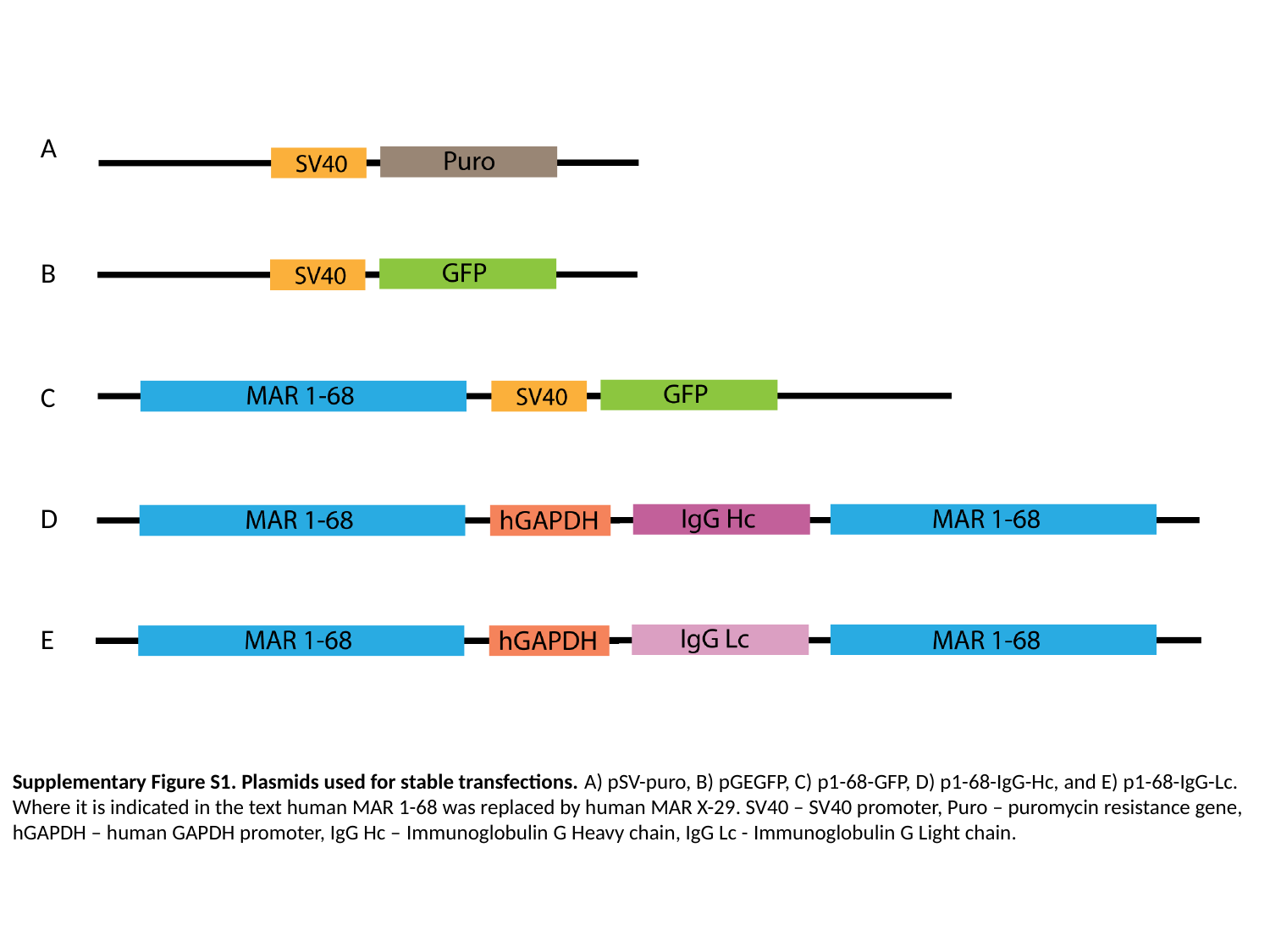

A
B
C
D
E
Supplementary Figure S1. Plasmids used for stable transfections. A) pSV-puro, B) pGEGFP, C) p1-68-GFP, D) p1-68-IgG-Hc, and E) p1-68-IgG-Lc. Where it is indicated in the text human MAR 1-68 was replaced by human MAR X-29. SV40 – SV40 promoter, Puro – puromycin resistance gene, hGAPDH – human GAPDH promoter, IgG Hc – Immunoglobulin G Heavy chain, IgG Lc - Immunoglobulin G Light chain.

## Slide 2
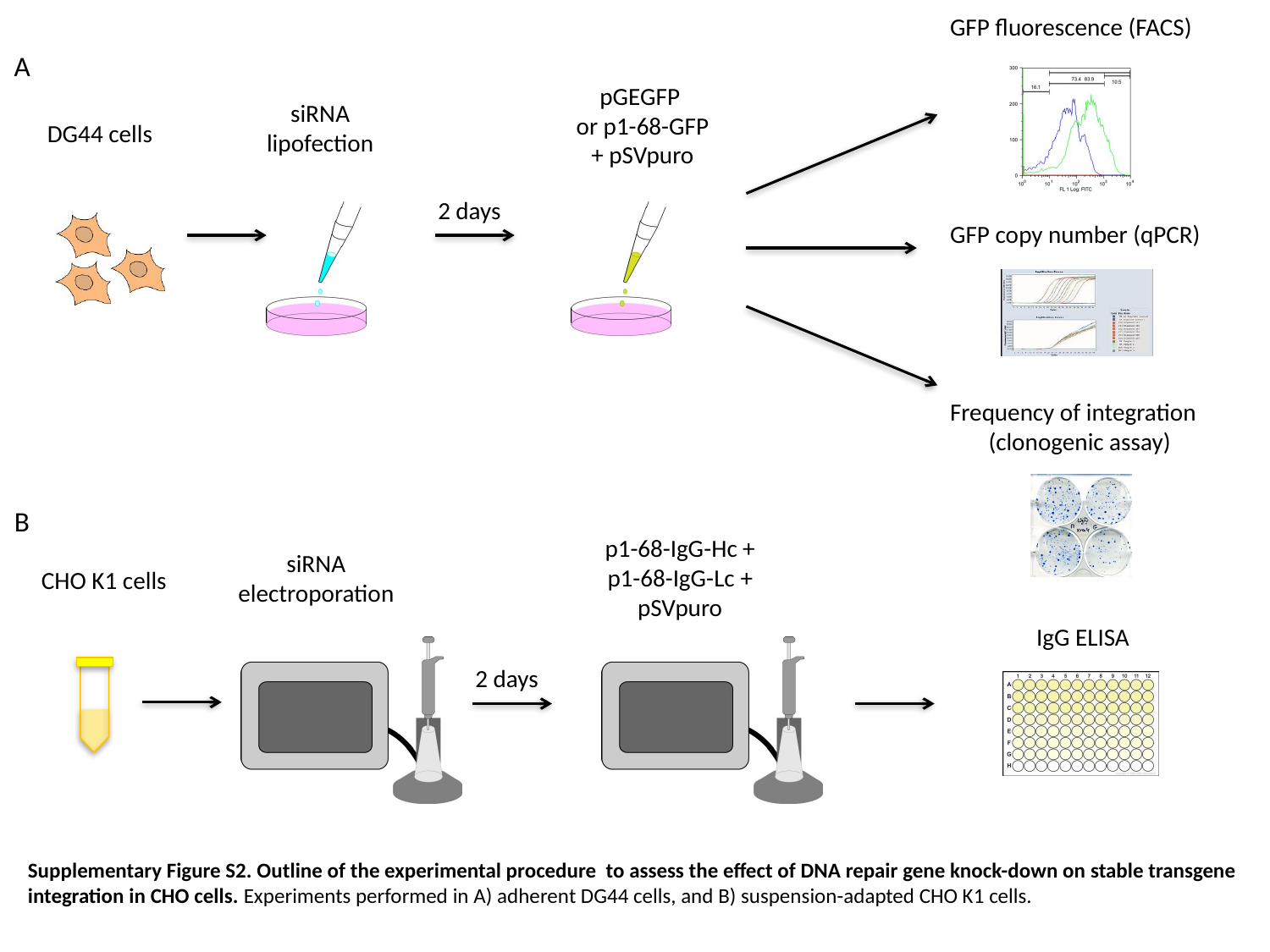

GFP fluorescence (FACS)
A
pGEGFP
or p1-68-GFP
+ pSVpuro
siRNA lipofection
DG44 cells
2 days
GFP copy number (qPCR)
Frequency of integration
 (clonogenic assay)
B
p1-68-IgG-Hc + p1-68-IgG-Lc + pSVpuro
siRNA
electroporation
CHO K1 cells
IgG ELISA
2 days
Supplementary Figure S2. Outline of the experimental procedure to assess the effect of DNA repair gene knock-down on stable transgene integration in CHO cells. Experiments performed in A) adherent DG44 cells, and B) suspension-adapted CHO K1 cells.

## Slide 3
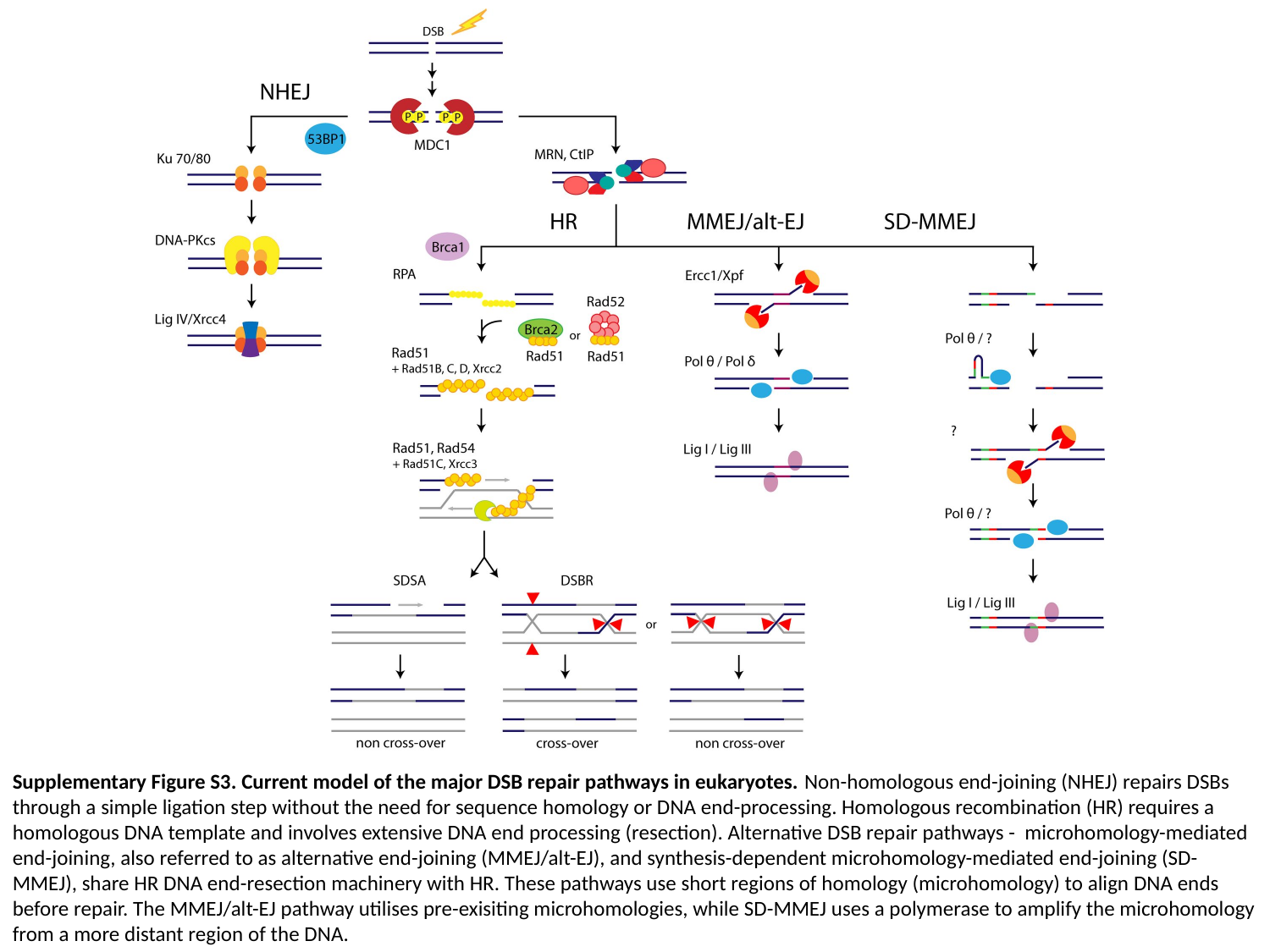

Introduction
Supplementary Figure S3. Current model of the major DSB repair pathways in eukaryotes. Non-homologous end-joining (NHEJ) repairs DSBs through a simple ligation step without the need for sequence homology or DNA end-processing. Homologous recombination (HR) requires a homologous DNA template and involves extensive DNA end processing (resection). Alternative DSB repair pathways - microhomology-mediated end-joining, also referred to as alternative end-joining (MMEJ/alt-EJ), and synthesis-dependent microhomology-mediated end-joining (SD-MMEJ), share HR DNA end-resection machinery with HR. These pathways use short regions of homology (microhomology) to align DNA ends before repair. The MMEJ/alt-EJ pathway utilises pre-exisiting microhomologies, while SD-MMEJ uses a polymerase to amplify the microhomology from a more distant region of the DNA.

## Slide 4
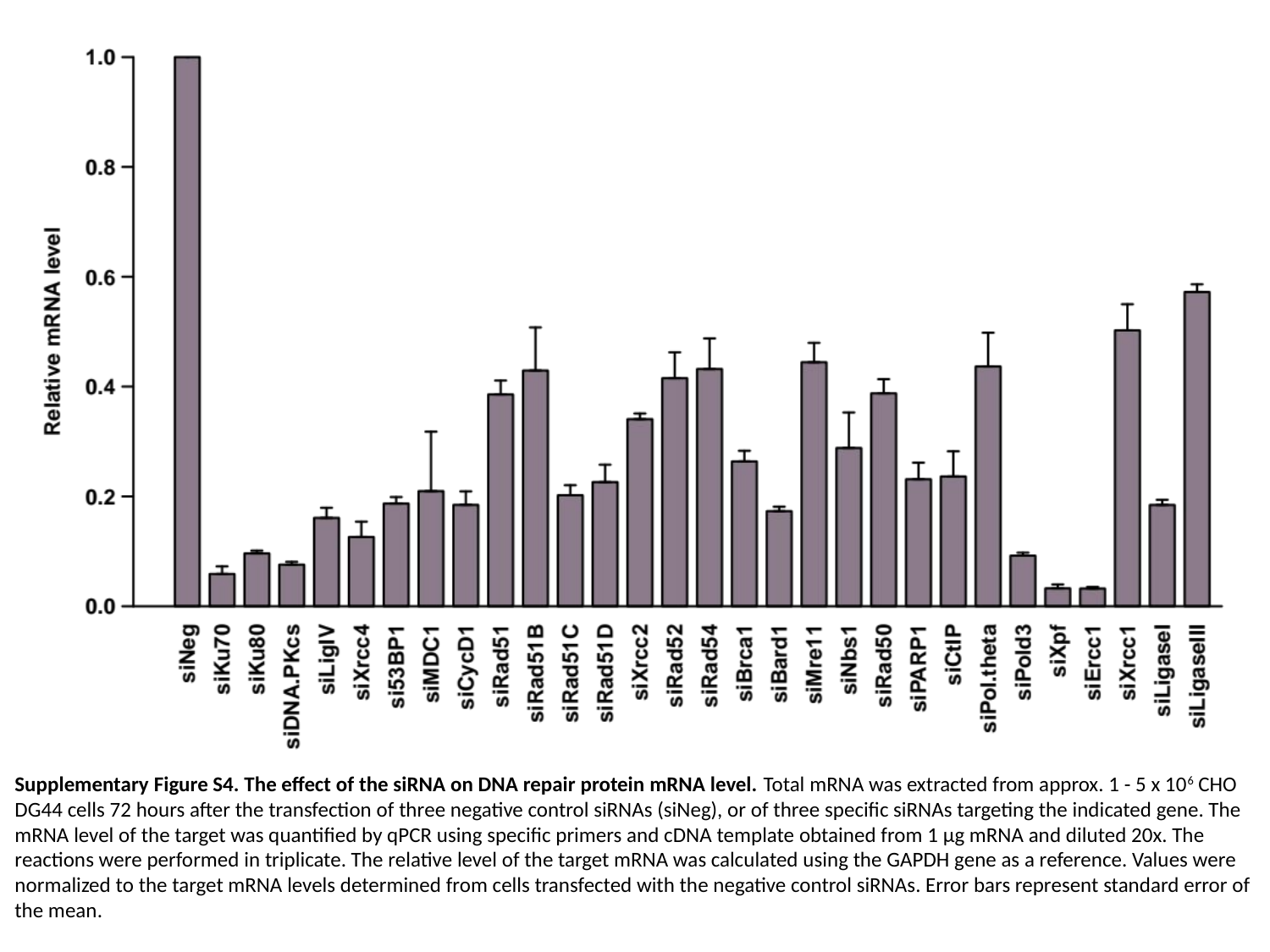

Supplementary Figure S4. The effect of the siRNA on DNA repair protein mRNA level. Total mRNA was extracted from approx. 1 - 5 x 10^6^ CHO DG44 cells 72 hours after the transfection of three negative control siRNAs (siNeg), or of three specific siRNAs targeting the indicated gene. The mRNA level of the target was quantified by qPCR using specific primers and cDNA template obtained from 1 µg mRNA and diluted 20x. The reactions were performed in triplicate. The relative level of the target mRNA was calculated using the GAPDH gene as a reference. Values were normalized to the target mRNA levels determined from cells transfected with the negative control siRNAs. Error bars represent standard error of the mean.

## Slide 5
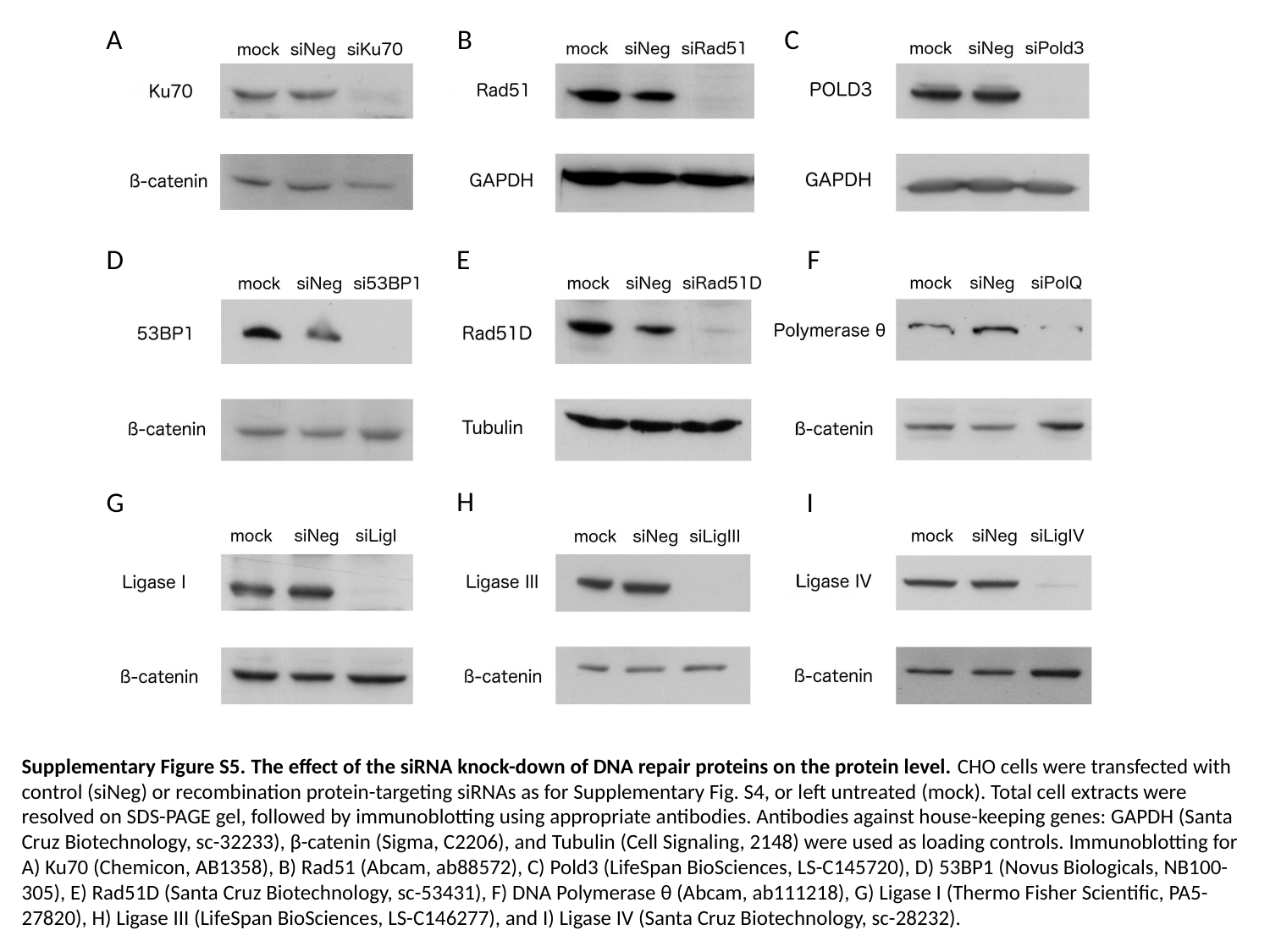

A
B
C
D
E
F
H
G
I
Supplementary Figure S5. The effect of the siRNA knock-down of DNA repair proteins on the protein level. CHO cells were transfected with control (siNeg) or recombination protein-targeting siRNAs as for Supplementary Fig. S4, or left untreated (mock). Total cell extracts were resolved on SDS-PAGE gel, followed by immunoblotting using appropriate antibodies. Antibodies against house-keeping genes: GAPDH (Santa Cruz Biotechnology, sc-32233), β-catenin (Sigma, C2206), and Tubulin (Cell Signaling, 2148) were used as loading controls. Immunoblotting for A) Ku70 (Chemicon, AB1358), B) Rad51 (Abcam, ab88572), C) Pold3 (LifeSpan BioSciences, LS-C145720), D) 53BP1 (Novus Biologicals, NB100-305), E) Rad51D (Santa Cruz Biotechnology, sc-53431), F) DNA Polymerase θ (Abcam, ab111218), G) Ligase I (Thermo Fisher Scientific, PA5-27820), H) Ligase III (LifeSpan BioSciences, LS-C146277), and I) Ligase IV (Santa Cruz Biotechnology, sc-28232).

## Slide 6
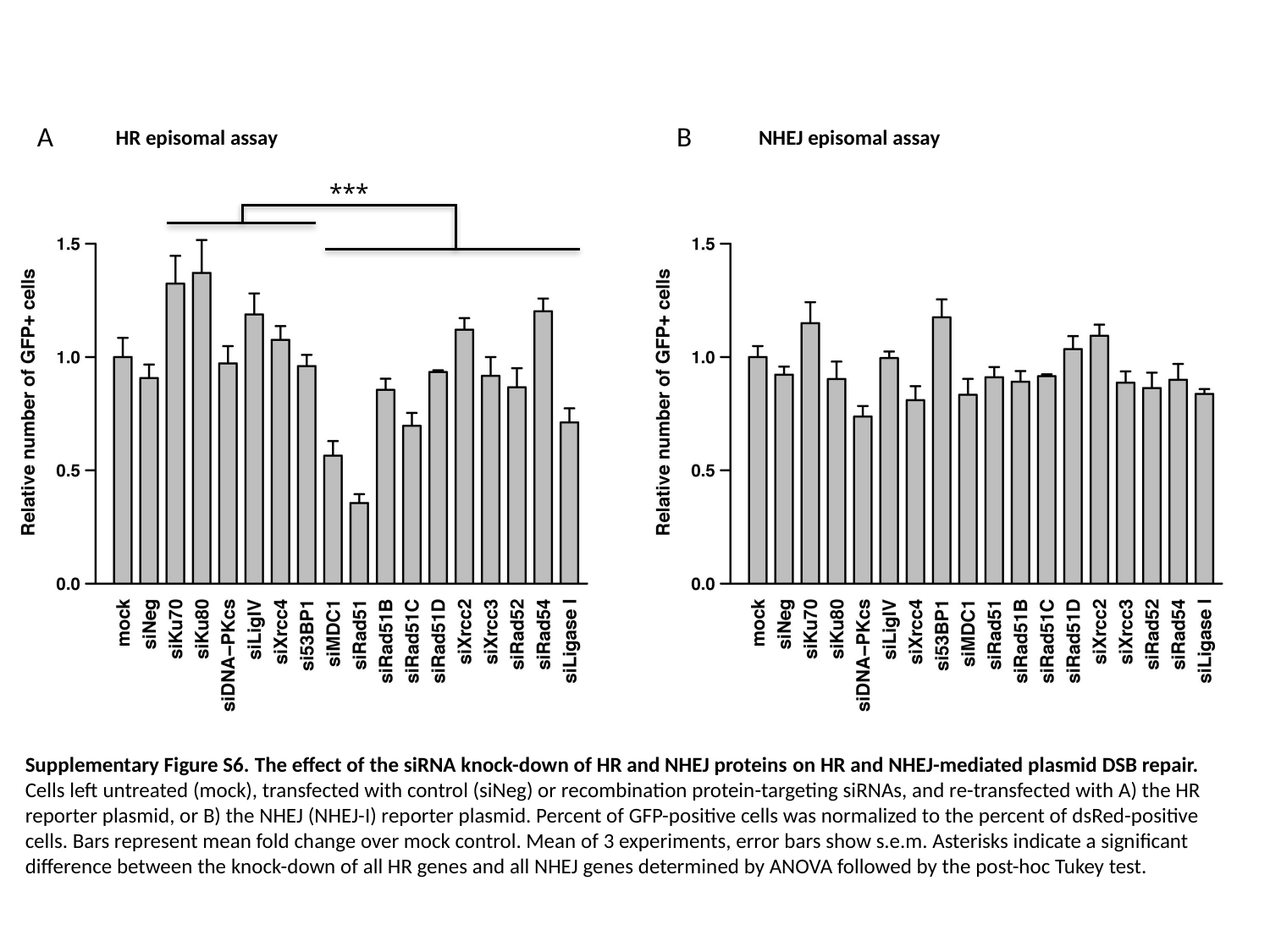

A
B
HR episomal assay
NHEJ episomal assay
***
Supplementary Figure S6. The effect of the siRNA knock-down of HR and NHEJ proteins on HR and NHEJ-mediated plasmid DSB repair. Cells left untreated (mock), transfected with control (siNeg) or recombination protein-targeting siRNAs, and re-transfected with A) the HR reporter plasmid, or B) the NHEJ (NHEJ-I) reporter plasmid. Percent of GFP-positive cells was normalized to the percent of dsRed-positive cells. Bars represent mean fold change over mock control. Mean of 3 experiments, error bars show s.e.m. Asterisks indicate a significant difference between the knock-down of all HR genes and all NHEJ genes determined by ANOVA followed by the post-hoc Tukey test.

## Slide 7
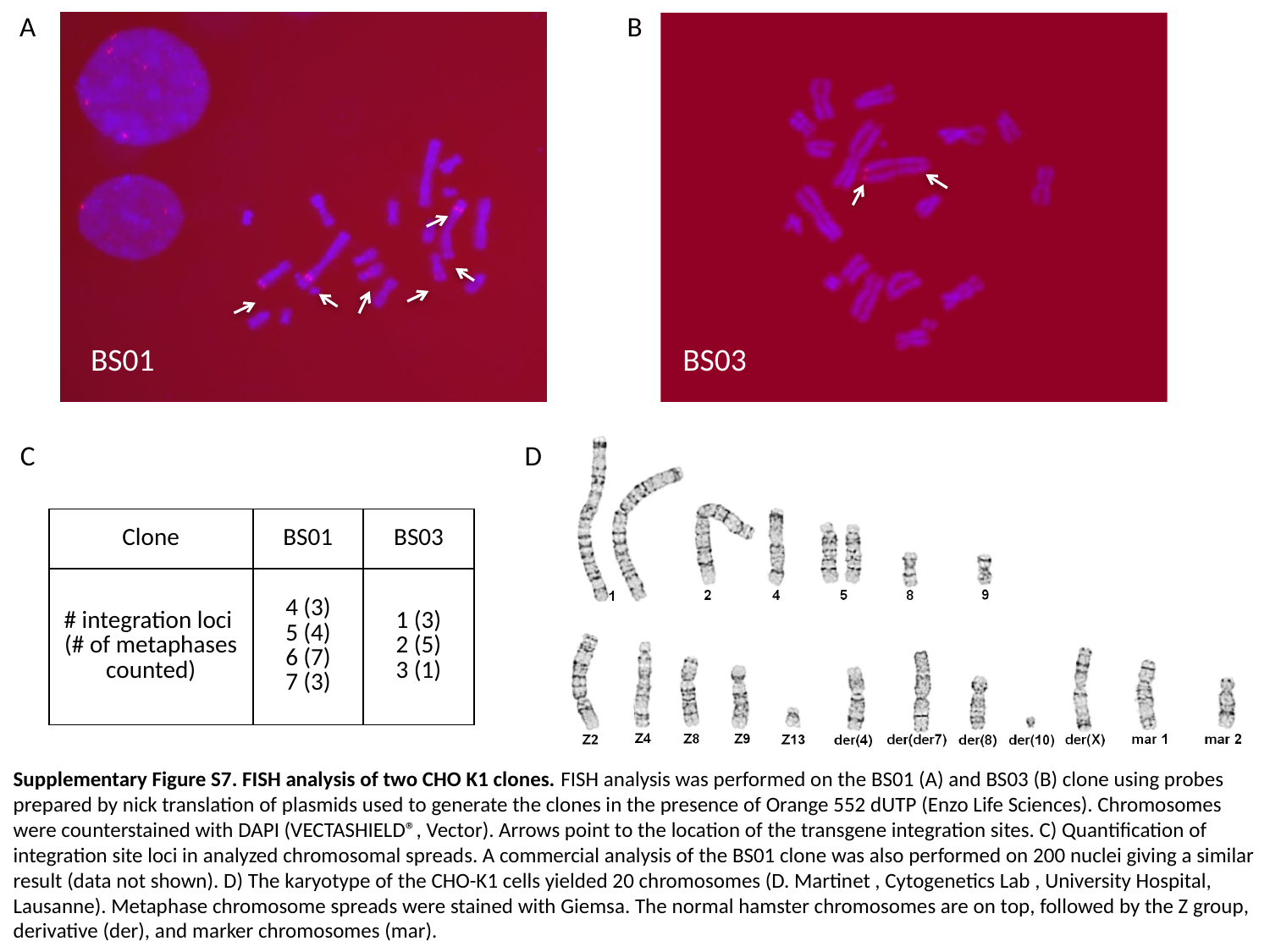

A
B
BS01
BS03
C
D
| Clone | BS01 | BS03 |
| --- | --- | --- |
| # integration loci (# of metaphases counted) | 4 (3) 5 (4) 6 (7) 7 (3) | 1 (3) 2 (5) 3 (1) |
Supplementary Figure S7. FISH analysis of two CHO K1 clones. FISH analysis was performed on the BS01 (A) and BS03 (B) clone using probes prepared by nick translation of plasmids used to generate the clones in the presence of Orange 552 dUTP (Enzo Life Sciences). Chromosomes were counterstained with DAPI (VECTASHIELD®, Vector). Arrows point to the location of the transgene integration sites. C) Quantification of integration site loci in analyzed chromosomal spreads. A commercial analysis of the BS01 clone was also performed on 200 nuclei giving a similar result (data not shown). D) The karyotype of the CHO-K1 cells yielded 20 chromosomes (D. Martinet , Cytogenetics Lab , University Hospital, Lausanne). Metaphase chromosome spreads were stained with Giemsa. The normal hamster chromosomes are on top, followed by the Z group, derivative (der), and marker chromosomes (mar).

## Slide 8
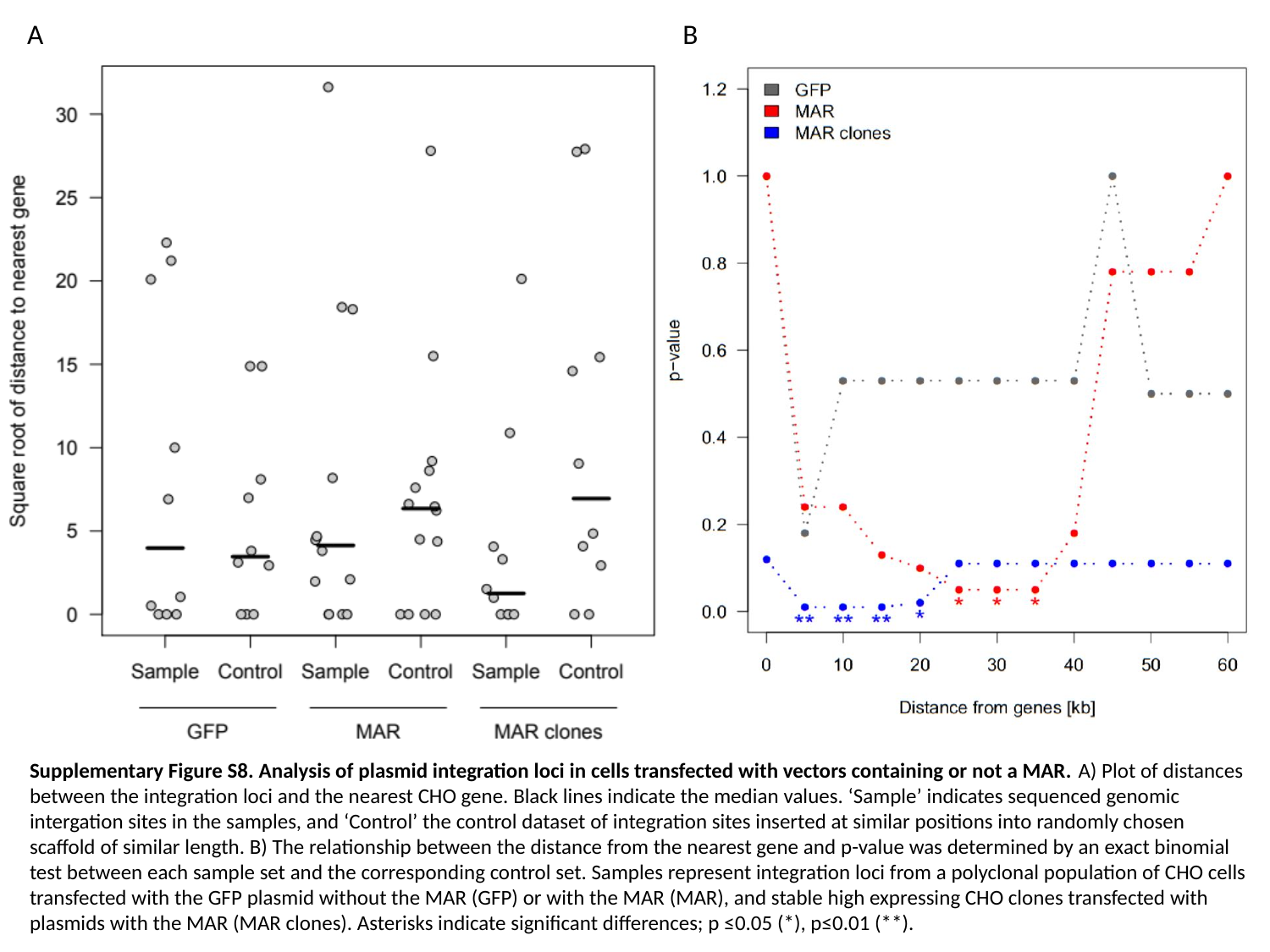

A
B
Supplementary Figure S8. Analysis of plasmid integration loci in cells transfected with vectors containing or not a MAR. A) Plot of distances between the integration loci and the nearest CHO gene. Black lines indicate the median values. ‘Sample’ indicates sequenced genomic intergation sites in the samples, and ‘Control’ the control dataset of integration sites inserted at similar positions into randomly chosen scaffold of similar length. B) The relationship between the distance from the nearest gene and p-value was determined by an exact binomial test between each sample set and the corresponding control set. Samples represent integration loci from a polyclonal population of CHO cells transfected with the GFP plasmid without the MAR (GFP) or with the MAR (MAR), and stable high expressing CHO clones transfected with plasmids with the MAR (MAR clones). Asterisks indicate significant differences; p ≤0.05 (*), p≤0.01 (**).

## Slide 9
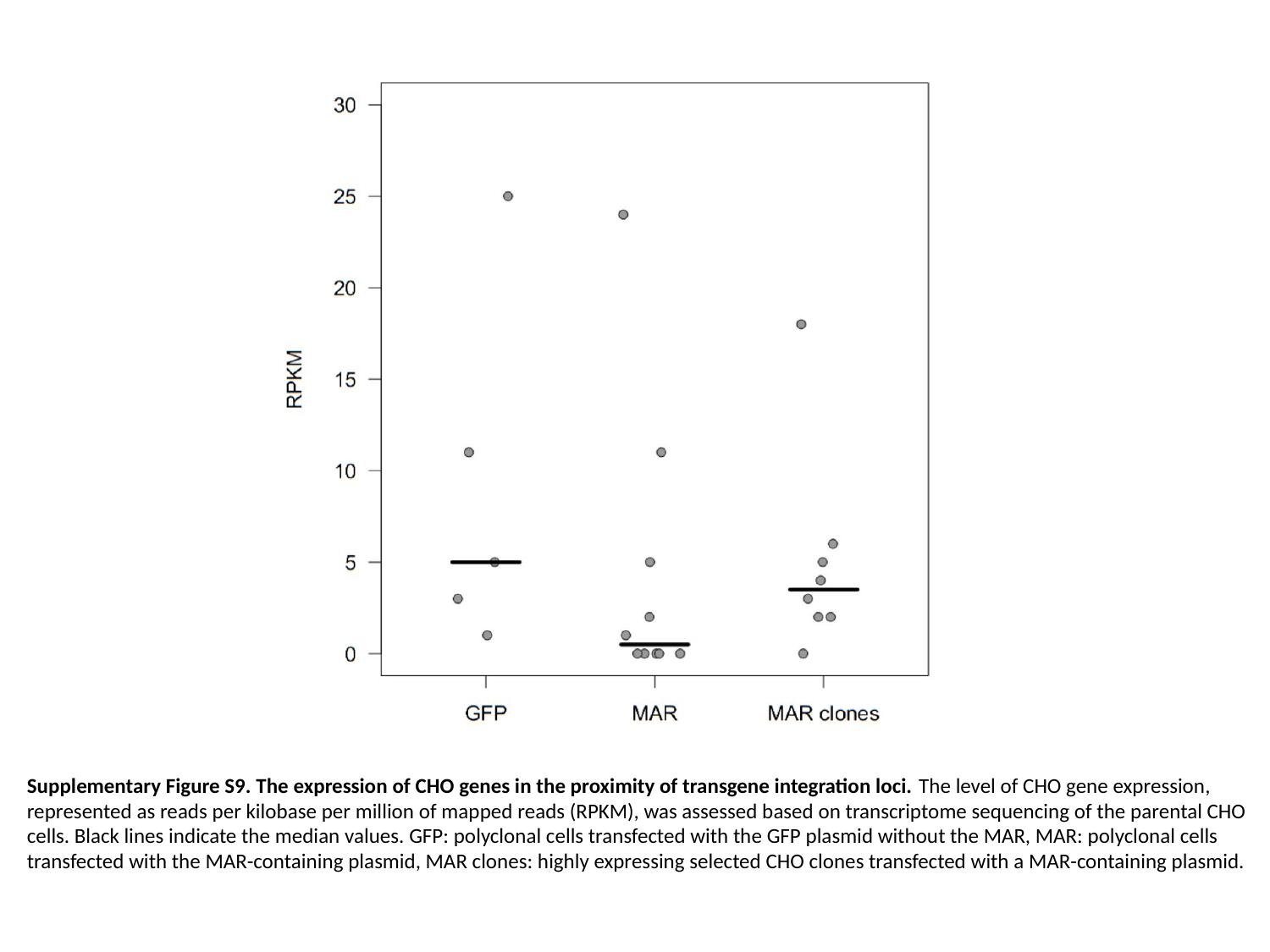

Supplementary Figure S9. The expression of CHO genes in the proximity of transgene integration loci. The level of CHO gene expression, represented as reads per kilobase per million of mapped reads (RPKM), was assessed based on transcriptome sequencing of the parental CHO cells. Black lines indicate the median values. GFP: polyclonal cells transfected with the GFP plasmid without the MAR, MAR: polyclonal cells transfected with the MAR-containing plasmid, MAR clones: highly expressing selected CHO clones transfected with a MAR-containing plasmid.
