# Supplementary material for: MAR‐Mediated transgene integration into permissive chromatin and increased expression by recombination pathway engineering
Source: Biotechnol Bioeng. 2016 Oct 3;114(2):384–96. doi: 10.1002/bit.26086 (PMC5215416; doi:10.1002/bit.26086)
Supplement: Supplementary file 2 — Supplementary Tables [file BIT-114-384-s002.pdf]

Supplementary Table S1. List of HR and NHEJ targets for siRNA knock-down.

Pathway	Target	References
NHEJ	Ku70/Ku80	Walker et al., 2001
	DNA-PKcs	Allen et al., 2002; Dobbs et al., 2010; Sipley et al., 1995
	LigIV	Critchlow et al., 1997
	Xrcc4	Critchlow et al., 1997; Li et al., 1995
	53BP1	Xie et al., 2007
HR	MDC1	Stucki et al. 2005; Zhang et al. 2005; Lou et al. 2006; Xie et al. 2007
	Rad51	Benson et al. 1994; Baumann and West 1998; Vispé et al. 1998; Arnaudeau et al. 1999
	Rad51B, Rad51C, Rad51D, Xrcc2, Xrcc3	Cartwright et al. 1998; Pittman et al. 1998; Masson et al. 2001; Takata et al. 2001; Lio et al. 2004; Liu et al. 2004; Suwaki et al. 2011
	Rad52	Van Dyck et al. 1998; Van Dyck et al. 1999; Feng et al. 2011
	Rad54	Essers et al. 2002; Heyer et al. 2006
	Brca1	Yoshida and Miki 2004; Cousineau et al. 2005
	Bard1	Wu et al. 1996
	Brca2	Davies et al. 2001; Moynahan et al. 2001; Yoshida and Miki 2004; Liu et al. 2010; Feng et al. 2011
	MRN (MRX in yeast)	Stracker and Petrini 2011
	CtIP	Yu and Chen 2004; Sartori et al. 2007; You et al. 2009
	Cyclin D1	Li et al. 2010; Jirawatnotai et al. 2011

Supplementary Table S2. List of MMEJ targets for siRNA knock-down.

**Target**	**References**
MRN (MRX in yeast)	Ma et al. 2003; Zhang and Paull 2005; Lee and Lee 2007; Dinkelmann et al. 2009; Della-Maria et al. 2011
CtIP	Yun and Hiom 2009; Wang et al. 2012
PARP1	Audebert et al. 2004
Ercc1/Xpf (Rad1/Rad10 in yeast)	Ma et al. 2003; Lee and Lee 2007
Ligase I	Liang et al. 2008; Crespan et al. 2012; Paul et al. 2013; Oh et al. 2014
Ligase III (absent in yeast)	Audebert et al. 2004; Liang et al. 2008; Della-Maria et al. 2011; Paul et al. 2013; Oh et al. 2014
Xrcc1 (absent in yeast)	Della-Maria et al. 2011
DNA Polymerase delta subunit 3 (POLD3) (POL32 in yeast)	Lee and Lee 2007; Costantino et al. 2014
DNA Polymerase theta (POLQ) (absent in yeast)	Yu and McVey 2010; Koole et al. 2014; Kent et al. 2015

Supplementary Table S3. siRNA sequences.

Target gene	Name and sequence of the oligo sense strand
Negative control	Neg_1 AGGUAGUGUAAUCGCCUUG<br>Neg_2 GACGACUCACAUACGUAAA<br>Neg_3 GAAUAUAUCGCGAAAUGUA
Ku70	Ku70_1 GGUGCCCUUUACUGAGAAA<br>Ku70_2 AAAGCCCAAGGUAGAGUUA<br>Ku70_3 ACAUUUCCAAGACACAAUU
Ku80	Ku80_1 GAAACUGUCUAUUGCUUAA<br>Ku80_2 CCAUAGGGAAGAAGUUUGA<br>Ku80_3 GGAUUCCUAUGAGUGUUUA
DNA-PKcs	DNA-PKcs_1 GGAUCGAGCUGUUCAGAAA<br>DNA-PKcs_2 AGAUGAUGUUCACUCUAAA<br>DNA-PKcs_3 AUCCAUCGGUAUCUUUAAA
DNA Ligase IV	LigIV_1 AGAGCCUCCUUCAGUUAAU<br>LigIV_2 CUAUACAGCAGGUAAAUGA<br>LigIV_3 AGAGGUAUGAUAUCCUUAA
Xrcc4	Xrcc4_1 AUAUGCUGAUGAAUUGAGA<br>Xrcc4_2 CUGAAAGAUGUCUCAUUUA<br>Xrcc4_3 AUGAGCACCUGCAGAAAGA
53BP1	53BP1_1 UCAGAAUGAUGACAAAGUA<br>53BP1_2 GAGCAAGGAGACAAUAAUA<br>53BP1_3 CAAAGACAUCCCUGUUACA
Cyclin D1	CycD1_1 UGGAACUCCUUCUGGUGAA<br>CycD1_2 CGCACUUUCUUUCCAGAGU<br>CycD1_3 UGCCAGAGGCGGAUGAGAA
MDC1	MDC1_1 ACAGCAUGCAGUAAUUGAA<br>MDC1_2 GGAAGAAGAUCCUGAGGAA<br>MDC1_3 CACGGAAAUGGGUGAAGAA
Rad51	Rad51_1 GUGCCAAUGAUGUGAAGAA<br>Rad51_2 GGGAAUUAGUGAAGCCAAA<br>Rad51_3 GGCGUUCAGAAAUCAUACA
Rad51B	Rad51b_1 ACAGCCUAUGAUAUAAAGA<br>Rad51b_2 CAAGUUCUUGGCCAAACAA<br>Rad51b_3 GUACCUGGCUGAGGAAUUU
Rad51C	Rad51c_1 UGAUCAGCCUGGCAAAUAA<br>Rad51c_2 AGAGGAAGCUUUAGAAACU<br>Rad51c_3 GGAUGAAGAACACCAGAAA
Rad51D	Rad51d_1 ACGGAGCAGACCUAUAUGA<br>Rad51d_2 CCCAAGAUGAGGAGAAACA<br>Rad51d_3 GCCUGGACAAACUACUUGA
Xrcc2	Xrcc2_1 GAAGUGUUCUCAGCUCCUA<br>Xrcc2_2 CAACACAAAGUCUAAUGCA<br>Xrcc2_3 AUCAGAGGGUGGACUGCAA

Supplementary Table S3. siRNA sequences (continued).

Rad52	Rad52_1 UGAGAUGUUUGGUUACAAU<br>Rad52_2 ACUGCAUUCUGGACAAAGA<br>Rad52_3 CCCUGAAGACAACCUUGAA
Rad54	Rad54_1 AGAAGACCUGCUAUAUUUA<br>Rad54_2 CAUCAGAUAUCCUCUCUAA<br>Rad54_3 GAAGCUAUGUAACCAUCCA
Brca1	Brca1_1 CCACGUAACUGAAAUUAUA<br>Brca1_2 AAGGCUGAGUUCUAUAAUA<br>Brca1_3 AGAGCCAAAUGAACAAAGA
Bard1	Bard1_1 GAACGGCCAUGUGGAUAUA<br>Bard1_2 ACAGACAAUUGGACAACAU<br>Bard1_3 GCAGCAGAAGAAAUCUUUA
Mre11	Mre11_1 AGAUGCAGUUCGAGGAAAU<br>Mre11_2 AAACAGGUGAAGAGAUCAA<br>Mre11_3 UUACUCAGAGACUAUUGAA
Nbs1	Nbs1_1 GAAACAGCCUCCAGAAAUU<br>Nbs1_2 CAAUUGAUUUGGCUAUAGA<br>Nbs1_3 AAACUGUGCCAUUCUGAUA
Rad50	Rad50_1 UAAUGAGACUUGACAAUGA<br>Rad50_2 ACACUCUUGGGUACAAUAA<br>Rad50_3 ACAGAACUCCUCACUAAGA
CtIP	CtIP_1 GUGCAAGGUUUACAAAUAA<br>CtIP_2 CAAAGUCCCUGCCAAACAA<br>CtIP_3 AGAAUACUCUCCAGGAAGA
PARP1	PARP1_1 AGGAGUUGCUUAUCUUCAA<br>PARP1_2 UAUCCUACCUCAAGAAAUU<br>PARP1_3 UGACACCUGCCUACUAUAU
DNA polymerase θ	Poltheta_1 UCAGUGAAAUUCCCUUAAA<br>Poltheta_2 GUUCAGGGCUACUUAUAAA<br>Poltheta_3 CGAAAGGCCUAAAUUAACA
Pold3	POLD3_1 AACAGAUGCUCUAUGAAUA<br>POLD3_2 CCAAAGCAGAGGCUAAAGA<br>POLD3_3 ACGAAAGCGUGUACUGAAA
Xpf1	Xlf1_1 GUAGAAUACUUGUGGUUGA<br>Xlf1_2 GAACCCUACUGCAGUAUCU<br>Xlf1_3 GAAGUGUGGGUGAAUCUUA
Ercc1	Ercc1_1 ACGGGAGCGAAAUCCAAUA<br>Ercc1_2 GUUUGUGAUCCCACUGGAA<br>Ercc1_3 GCCCUUAUUCAGAUCCUCA
Xrcc1	Xrcc1_1 GCGCUGGGACCGUGUUAAA<br>Xrcc1_2 ACUGGACUUGAAUCUAGAA<br>Xrcc1_3 GCUUAUCCGAUACGUUACA
DNA Ligase I	Ligase 1_1 UUACAAUCCUUCCAAGAGA<br>Ligase 1_2 AGACAUGGUUGGAAGAACA<br>Ligase 1_3 AAGGAAGAAGGAAGAAGAA
DNA Ligase III	LigIII_1 UCACUGGCCUGUCAUAAGA<br>LigIII_2 GCACAAAGACUGUCUACUA<br>LigIII_3 AAUCCUAGCUACAAUACAA

Supplementary Table S4. Sequences of the DNA junctions of Immunoglobulin expression vector genomic integration sites (part 1).

Clone	Integration site #	Junction side		Sequence[1]	Mechanism[2]	Integration in/near a gene[3] expressed (yes/no)	Deletion in the genome (size)	Templated insert[4] (size)
BS01	1	Left	junction	GCGAGCAGAACGGAGACTGAAGGGGTGGGGCCGCGGCCGACAATGGGCGGGGGTCGTTGGGCGGTCAGCCAGGCGGGCCATTTACCGTAAGTTATGTAA CGCGGAATGGCGAATGGCAAATTGTAAGCGTTAATATTTTGTTAAAATTCGCGTTAAATTTTTGTTAAATCAGCTCATTTTTTAACCAATA	SD-MMEJ (5 nt)	Ssh3 (exon), yes (RPKM=3)	no	yes (66bp)
			genome	...CTATTGTCCTTGCTC(206bp)TGGGGCCGCGGCCGATCGG...	(IR and DR)			
			vector frgm 1	...TTATTGACGTCAATGGGCGGGGGTCGTTGGGCGGTCAGCCAGGCGGGCCATTTACCGTAAGTTATG TAACGCGGAACTCCATATATGGGCTATGA...	MMEJ (3 nt)			
			vector frgm 2	...CCTGAATGGCGAATGGCAAATTGTAAGCGTTAATATTTTGTTAAAATTCGCGTTAAATTTTTGTTAAATCAGCTCATTTTTTAA...				
		Right	junction	AAAATACAAAAATTAGCCAGGTGTGTCGAATGGACAACCCGAGAATAACTATAAGACGACGCCACCCGTCCTTGATAGCGACGGTTCCTTTTTCTTGTA CAGCAAACTGACGGTGGACAAATCAAGATCGGTGCGAGCTCTGTGGGGCCGGACTGGGCACCAGTC	SD-MMEJ (4 nt)			yes (100bp)
			vector frgm 1	...ACCCTGTCTC(24bp)GGTGTGGTGG...	(DR)			
			vector frgm 2	...AGACATTGCGGTAGAGTGGGAGTCGAATGGACAACCCGAGAATAACTATAAGACGACGCCACCCGTCCTTGATAGCGACGGTTCCTTTTTCTTGTA CAGCAAACTGACGGTGGACAAATCAAGATGGCAGC...	MMEJ (3 nt)			
			genome	...GGCCGATCGGTGCGAGCTCTGTGGGGCCGGACTGGGCACCAGTCGGGCCCTTTAAGGGCCGCTCCCGA...				
	2	Left	junction	AAAATCCCCGCCCTTTGCTGGCGGCTCGGCGGTCGATGATGCCTCTTCGCTATTACGCCAGCCCAAGCTACCATGATAAG	SD-MMEJ (10 nt)	Dph1 (exon), yes (RPKM=18)	no	yes (3bp)
			genome	...ATCCCCGCCCTTTGCTGGCGGCTCGGCGGTCGATCG...				
			vector	...ATCGGTGCGGGCCTCTTCGCTATTA(1103bp)CTTGGTTGACGGCAATTTCGATGATGCAGC...				
		Right	junction	TTAAGGTAATCTTAAGTAGAAGAGATAGAGTTTAGAATTTTTTAATCGTGCTGCCTGGGTTCTCCGCGTTCCTCCAGCGC	SD-MMEJ (5 nt)			no
			vector	...AGGTAATCTTAAGTAGAAGAGATAGAGTTTAGAATTTTTTAAATTTATCTCT...	(DR)			
			genome	...CGATCGTGCTGCCTGGGTTCTCCGCGTTCCTCCAGCGCTGCCTTTTTGGTC...				
	3	Left	junction	AAATTATACTGAGTAAGGTAACTCTGGACCAGAAATGAGAATCGACCGATGCCCTTGAGAGCCTTCAACCCAGTCAGCTC	MMEJ (2nt)	intergenic (404.9kb from nearest gene)	n.a.[5]	no
			genome	...TTATACTGAGTAAGGTAACTCTGGACCAGAAATGAGAATGACCTGTGTT...	SD-MMEJ (4 nt)			
			vector	...CAGCAGCATAGGGATCCGTCGATCGACCGATGCCCTTGAG(25bp)CGGTGGGCGCGGGGCATGACTATCGTCG...	(IR or DR)			
	4	Left	junction	GTCCCTACCCGCCTCCTGGCGCTGGTCTCCGACTTGGAGCGGCCCTGCATCTCAATTAGTCAGCAACCATAGTCCCGCCC	SD-MMEJ (4 nt)	10.9kb from lin-54 homolog, yes (RPKM=4)	n.a.	no
			genome	...CCTACCCGCCTCCTGGCGCTGGTCTCCGATCGGTCCTCCAGAGCGCGCG...	(IR or DR)			
			vector	...ACGGGAGGTACTTGGAGCGGCCCTGCATCTCAATTAGTCAGCAAC(68bp)ATCGCTGACTAATTTTTTTT...				
	5	Right	junction	ATGGTAGCTTGGGCTGGCGTAATAGCGAAGAGGCCCGCACCGGCGGGCCAATTTCTTTATGCATATCGCTGTGTCCTAGG	SD-MMEJ (6 nt)	Ankzf1 (exon), yes (RPKM=2)	n.a.	yes (2bp)
			vector	...TGGGCGGAGTTAGGGGCGGGACTATGGT(103bp)ACCGATCGCCCTT...	(DR)			
			genome	...TTTTCAAAAATGCCCAGGGCCAATTTCTTTATGCATATCGCTGTGTCCTAGGCCCTCGACAGGCA...				
	6	Right	junction	ACATTCGACGATTATGCGATGCATTGGGTTCGTCAGATGAGGCTCTACAAGGTGAAATGTCTTCTTGCAGAGCCTCTCTA	MMEJ (4 nt)	16.5kb from Amigo1, no (RPKM=0)	n.a.	no
			vector	...TTCGACGATTATGCGATGCATTGGGTTCGTCAGGCGCCTGGGAAGGGTC...	SD-MMEJ (6 nt)			
			genome	...TACTCTTTCGAGGGCTCAGTCCTATCAGATGAGGCTCTACAAGGTGAAATGTC(76bp)CTCTCTGACACGACTACCTCTTTCTC...	(IR)			

Supplementary Table S4. Sequences of the DNA junctions of Immunoglobulin expression vector genomic integration sites (part 2).

Clone	Integration site #	Junction side		Sequence[1]	Mechanism[2]	Integration in/near a gene[3] expressed (yes/no)	Deletion in the genome (size)	Templated insert[4] (size)
**BS03**	**1**	**Left**	**junction**	TAATGCGTCCTTGCTTTGCTCTGAGCGCTTCTTGTCTCGAACAGGGGGGGTGGTCTTGTAGTTGTTCTCGGGCTGTCCGT	SD-MMEJ (5 nt)	**17.9kb from Bahcc1,** yes (RPKM=5)	yes (~913 bp)	no
			**genome**	...TGCGTCCTTGCTTTGCTCTGAGCGCTTCTTGTCTCGATCGACACCCT...	(IR)			
			**vector**	...GGAGTCCAGCACAGGGGGGGTGGTCTTGTAGTTGTTCTCGGGCTGTCCGTTGGACTCCCATTC...				
		**Right**	**junction**	GGGGTGGGGTGGGGCAGGACAGCAAGGGGGAGGATTGGGAAGACAATAGCAGGGGGTGGGGAGCCAAGCCGCGGCGAGAGCGCAAAGCCCGCG	MMEJ (5nt)			no
			**vector**	...GTGGGGTGGGGCAGGACAGCAAGGGGGAGGATTGGGAAGACAATAGCAGGCCCACCCCAC...	SD-MMEJ (5 nt)			
			**genome**	...CGATCGGCCCGCAGGGGGTGGGGAGCCAAGCCGCGGCGAGAGCGCAAAGCCCGCG...	(DR)			
	**2**	**Right**	**junction**	GGCGAAGGGGCTCCTTAAGCGCAAGGCCTCGAACTCTCCCTCGCTTGAGTGCGGGGTCAGCTCAGGAAACCCTGTCTCTT	SD-MMEJ (5 nt)	**1kb from C17orf70,** yes (RPKM=6)	n.a.[5]	n.a.
			**vector**	...GAAGGGGCTCCTTAAGCGCAAGGCCTCGAACTCTCCCACCCACTTCC...	(DR)			
			**genome**	...TGGGGCACGATCGCTTGAGTGCGGGGTCAGCTCAGGAAACCCTGTCTCTTAAAAACCTTAAGC...				
**Cp33/64**	**1**	**Left**	**junction**	GCTTTTCTAACTTAAATTATCTGGTTTCTCTTTAACTACAATGCTGCTGGTTTACAGACCACATGTAGAGTGGCAATGTG	MMEJ (2nt)	**Cblb (intron),** yes (RPKM=2)	yes (320 bp)	no
			**genome**	...TTTCTAACTTAAATTATCTGGTTTCTCTTTAACTACAATTTGCCTCT...	SD-MMEJ (6 nt)			
			**vector**	...GGGCATCGGTCGATCGACGGATCCCTATGCTGCTGGTTTACAG(147bp)TTTAGCAAGACAGTGATAATGCTAATATG...	(DR)			
		**Right**	**junction**	TGCCATCCAGCACATTGCCACTCTACATGTGGTCTGTAAACCAGCAGCATACAGCATAAACAAATGTAACACAACTTAAA	SD-MMEJ (5 nt)			no
			**vector**	...TGAAATTACAATACAAATGATGAT(100bp)TGTAAACCAGCAGCATAGGGATCCGTCGAT...	(DR)			
			**genome**	...CAAATGTTCCACAGCATAAACAAATGTAACACAACTTAAACTAATATTTCACA...				
	**2**	**Left**	**junction**	GCCCTGCCACTTTGAACATACTTTCTTGTTTACTTATTACTATTTTCCTTTTATATGTTTAAATCATCATTTGTATTGTAATTTCATCTTCCA	SD-MMEJ (5 nt)	**intergenic** (118.4kb from nearest gene)	no	yes (1nt)
			**genome**	...CTGCCACTTTGAACATACTTTCTTGTTTACTTATTATGTTTATCCC...	(DR)			
			**vector**	...GAACAATTTTTATTTTCCTTTTATATGTTTAAATCATCATTTGTATTGTAATTTCATCTTCCA...				
		**Right**	**junction**	ACCTCCCGTACCTTAATATTACTTACTTATCATGGTAGCTTGTTTATCCCTTAGTCTTCCCACTCCTGACTTGAATGCTT	MMEJ (2nt)			no
			**vector**	...TCCCGTACCTTAATATTACTTACTTATCATGGTAGCTTGGGCTGGCG...	SD-MMEJ (4 nt)			
			**genome**	...TTACTTATTATGTTTATCCCTTAGTCTTCCCACTCCTGACTTGAATGCTTACTCTTTGAGGAG...	(DR)			

[1] Sequenced plasmid integration junctions are represented by blue (CHO genome) and green (vector sequence) letters, as predicted from whole genome sequencing of Illumina genomic and mate-pair libraries, and as validated experimentally by PCR amplification and direct sequencing of the junctions.
[2] This lists the most probable mechanisms accounting for the junction, consisting of the Microhomology-mediated end-joining (MMEJ) and/or synthesis-dependent MMEJ (SD-MMEJ). The length of the microhomology is indicated in parenthesis. IR and DR indicate the use of inverted or direct repeat as a template for SD-MMEJ, respectively.
[3] Genomic integration site locus. The gene nearest to the integration site is listed in bold. Gene expression was assessed by total RNA sequencing of the parental CHO cells. Reads per kilobase of transcript per million reads mapped (RPKM) are used as a measure of the mRNA level.
[4] Insertion of nucleotides templated from another part of the vector or genome. [5] n.a., not annotated

Supplementary Table S5. Analysis of plasmid integration sites in cells transfected with vectors containing or not the MAR element.

Sample[1]	Integration within genes[2]	Integration near genes[3]	Expressed genes[4]
Polyclonal population without MAR	7/14 (*)[5]	8/14	8/8
Polyclonal population with MAR	6/14	10/14 (*)	5/10
High expressing clones with MAR	6/10 (**)	8/10	7/8

[1] Polyclonal populations of CHO cells transfected with GFP or MAR-GFP plasmids were sequenced by high-throughput sequencing (Pacific Biosciences) and plasmid-to-genome junctions were predicted using bioinformatics tools. Integration sites in high expressing CHO clones transfected with MAR-containing plasmids were PCR-amplified and sequenced using Sanger sequencing.
[2] integration locus inside or within 5kb from an open reading frame (ORF)
[3] integration locus in a gene or within 35kb from a gene
[4] number of expressed genes in the neighborhood (within 35kb) of the integration locus
[5] Statistical significance calculated between each sample set and the corresponding control set using an exact binomial test. Significance levels $p \leq 0.05$ (*), $p \leq 0.01$ (**).

Supplementary References to Tables S1 and S2

1. Walker,J.R., Corpina,R.A. and Goldberg,J. (2001) Structure of the Ku heterodimer bound to DNA and its implications for double-strand break repair. *Nature*, **412**, 607–14.
2. Allen,C., Kurimasa,A., Brenneman,M.A., Chen,D.J. and Nickoloff,J.A. (2002) DNA-dependent protein kinase suppresses double-strand break-induced and spontaneous homologous recombination. *Proc. Natl. Acad. Sci. U. S. A.*, **99**, 3758–63.
3. Dobbs,T.A., Tainer,J.A. and Lees-Miller,S.P. (2010) A structural model for regulation of NHEJ by DNA-PKcs autophosphorylation. *DNA Repair (Amst).*, **9**, 1307–1314.
4. Sipley,J.D., Menninger,J.C., Hartley,K.O., Ward,D.C., Jackson,S.P. and Anderson,C.W. (1995) Gene for the catalytic subunit of the human DNA-activated protein kinase maps to the site of the XRCC7 gene on chromosome 8. *Proc. Natl. Acad. Sci. U. S. A.*, **92**, 7515–9.
5. Critchlow,S.E., Bowater,R.P. and Jackson,S.P. (1997) Mammalian DNA double-strand break repair protein XRCC4 interacts with DNA ligase IV. *Curr. Biol.*, **7**, 588–598.
6. Li,Z., Otevrel,T., Gao,Y., Cheng,H.-L., Seed,B., Stamato,T.D., Taccioli,G.E. and Alt,F.W. (1995) The XRCC4 gene encodes a novel protein involved in DNA double-strand break repair and V(D)J recombination. *Cell*, **83**, 1079–1089.
7. Xie,A., Hartlerode,A., Stucki,M., Odate,S., Puget,N., Kwok,A., Nagaraju,G., Yan,C., Alt,F.W., Chen,J., *et al.* (2007) Distinct roles of chromatin-associated proteins MDC1 and 53BP1 in mammalian double-strand break repair. *Mol. Cell*, **28**, 1045–57.
8. Lou,Z., Minter-Dykhouse,K., Franco,S., Gostissa,M., Rivera,M.A., Celeste,A., Manis,J.P., van Deursen,J., Nussenzweig,A., Paull,T.T., *et al.* (2006) MDC1 maintains genomic stability by participating in the amplification of ATM-dependent DNA damage signals. *Mol. Cell*, **21**, 187–200.
9. Stucki,M., Clapperton,J.A., Mohammad,D., Yaffe,M.B., Smerdon,S.J. and Jackson,S.P. (2005) MDC1 directly binds phosphorylated histone H2AX to regulate cellular responses to DNA double-strand breaks. *Cell*, **123**, 1213–26.
10. Zhang,J., Ma,Z., Treszezamsky,A. and Powell,S.N. (2005) MDC1 interacts with Rad51 and facilitates homologous recombination. *Nat. Struct. Mol. Biol.*, **12**, 902–9.
11. Arnaudeau,C., Helleday,T. and Jenssen,D. (1999) The RAD51 protein supports homologous recombination by an exchange mechanism in mammalian cells. *J. Mol. Biol.*, **289**, 1231–8.
12. Baumann,P. and West,S.C. (1998) Role of the human RAD51 protein in homologous recombination and double-stranded-break repair. *Trends Biochem. Sci.*, **23**, 247–51.
13. Benson,F.E., Stasiak,A. and West,S.C. (1994) Purification and characterization of the human Rad51 protein, an analogue of E.coli RecA. *EMBO J.*, **13**, 5764–5771.
14. Vispé,S., Cazaux,C., Lesca,C. and Defais,M. (1998) Overexpression of Rad51 protein stimulates homologous recombination and increases resistance of mammalian cells to ionizing radiation. *Nucleic Acids Res.*, **26**, 2859–64.
15. Cartwright,R., Dunn,A.M., Simpson,P.J., Tambini,C.E. and Thacker,J. (1998) Isolation of novel human and mouse genes of the recA/RAD51 recombination-repair gene family. *Nucleic Acids Res.*, **26**, 1653–9.
16. Lio,Y.-C., Schild,D., Brenneman,M.A., Redpath,J.L. and Chen,D.J. (2004) Human Rad51C deficiency destabilizes XRCC3, impairs recombination, and radiosensitizes S/G2-phase cells. *J. Biol. Chem.*, **279**, 42313–20.
17. Liu,Y., Masson,J.-Y., Shah,R., O'Regan,P. and West,S.C. (2004) RAD51C is required for Holliday junction processing in mammalian cells. *Science*, **303**, 243–6.
18. Masson,J.Y., Tarsounas,M.C., Stasiak,A.Z., Stasiak,A., Shah,R., McIlwraith,M.J., Benson,F.E. and West,S.C. (2001) Identification and purification of two distinct complexes containing the five RAD51 paralogs. *Genes Dev.*, **15**, 3296–307.
19. Pittman,D.L., Weinberg,L.R. and Schimenti,J.C. (1998) Identification, characterization, and genetic mapping of Rad51d, a new mouse and human RAD51/RecA-related gene. *Genomics*, **49**, 103–11.
20. Suwaki,N., Klare,K. and Tarsounas,M. (2011) RAD51 paralogs: roles in DNA damage signalling, recombinational repair and tumorigenesis. *Semin. Cell Dev. Biol.*, **22**, 898–905.
21. Takata,M., Sasaki,M., Tachiiri,S., Fukushima,T., Sonoda,E., Schild,D., Thompson,L.H. and Takeda,S. (2001) Chromosome instability and defective recombinational repair in knockout mutants of the five Rad51 paralogs. *Mol. Cell. Biol.*, **21**, 2858–2866.
22. Van Dyck,E., Hajibagheri,N.M., Stasiak,A. and West,S.C. (1998) Visualisation of human rad52 protein and its complexes with hRad51 and DNA. *J. Mol. Biol.*, **284**, 1027–38.
23. Van Dyck,E., Stasiak,A.Z., Stasiak,A. and West,S.C. (1999) Binding of double-strand breaks in DNA by human Rad52 protein. *Nature*, **398**, 728–731.
24. Feng,Z., Scott,S.P., Bussen,W., Sharma,G.G., Guo,G., Pandita,T.K. and Powell,S.N. (2011) Rad52 inactivation is synthetically lethal with BRCA2 deficiency. *Proc. Natl. Acad. Sci. U. S. A.*, **108**, 686–91.

25. Essers,J., Hendriks,R.W., Wesoly,J., Beerens,C.E.M.T., Smit,B., Hoeijmakers,J.H.J., Wyman,C., Dronkert,M.L.G. and Kanaar,R. (2002) Analysis of mouse Rad54 expression and its implications for homologous recombination. *DNA Repair (Amst).*, **1**, 779–93.
26. Heyer,W.-D., Li,X., Rolfsmeier,M. and Zhang,X.-P. (2006) Rad54: the Swiss Army knife of homologous recombination? *Nucleic Acids Res.*, **34**, 4115–25.
27. Cousineau,I., Abaji,C. and Belmaaza,A. (2005) BRCA1 regulates RAD51 function in response to DNA damage and suppresses spontaneous sister chromatid replication slippage: implications for sister chromatid cohesion, genome stability, and carcinogenesis. *Cancer Res.*, **65**, 11384–91.
28. Yoshida,K. and Miki,Y. (2004) Role of BRCA1 and BRCA2 as regulators of DNA repair, transcription, and cell cycle in response to DNA damage. *Cancer Sci.*, **95**, 866–71.
29. Wu,L.C., Wang,Z.W., Tsan,J.T., Spillman,M.A., Phung,A., Xu,X.L., Yang,M.-C.W., Hwang,L.-Y., Bowcock,A.M. and Baer,R. (1996) Identification of a RING protein that can interact in vivo with the BRCA1 gene product. *Nat. Genet.*, **14**, 430–40.
30. Davies,A.A., Masson,J.Y., McIlwraith,M.J., Stasiak,A.Z., Stasiak,A., Venkitaraman,A.R. and West,S.C. (2001) Role of BRCA2 in control of the RAD51 recombination and DNA repair protein. *Mol. Cell*, **7**, 273–82.
31. Liu,J., Doty,T., Gibson,B. and Heyer,W.-D. (2010) Human BRCA2 protein promotes RAD51 filament formation on RPA-covered ssDNA. *Nat. Struct. Mol. Biol.*, **17**, 1260–1262.
32. Moynahan,M.E., Pierce,A.J. and Jasin,M. (2001) BRCA2 is required for homology-directed repair of chromosomal breaks. *Mol. Cell*, **7**, 263–72.
33. Stracker,T.H. and Petrini,J.H.J. (2011) The MRE11 complex: starting from the ends. *Nat. Rev. Mol. Cell Biol.*, **12**, 90–103.
34. Sartori,A.A., Lukas,C., Coates,J., Mistrik,M., Fu,S., Bartek,J., Baer,R., Lukas,J. and Jackson,S.P. (2007) Human CtIP promotes DNA end resection. *Nature*, **450**, 509–14.
35. You,Z., Shi,L.Z., Zhu,Q., Wu,P., Zhang,Y.-W., Basilio,A., Tonnu,N., Verma,I.M., Berns,M.W. and Hunter,T. (2009) CtIP links DNA double-strand break sensing to resection. *Mol. Cell*, **36**, 954–69.
36. Yu,X. and Chen,J. (2004) DNA Damage-Induced Cell Cycle Checkpoint Control Requires CtIP , a Phosphorylation-Dependent Binding Partner of BRCA1 C-Terminal Domains. *Mol. Cell. Biol.*, **24**, 9478–9486.
37. Jirawatnotai,S., Hu,Y., Michowski,W., Elias,J.E., Becks,L., Bienvenu,F., Zagozdzon,A., Goswami,T., Wang,Y.E., Clark,A.B., *et al.* (2011) A function for cyclin D1 in DNA repair uncovered by protein interactome analyses in human cancers. *Nature*, **474**, 230–4.
38. Li,Z., Jiao,X., Wang,C., Shirley,L.A., Elsaleh,H., Dahl,O., Wang,M., Soutoglou,E., Knudsen,E.S. and Pestell,R.G. (2010) Alternative cyclin D1 splice forms differentially regulate the DNA damage response. *Cancer Res.*, **70**, 8802–11.
39. Della-Maria,J., Zhou,Y., Tsai,M.-S., Kuhnlein,J., Carney,J.P., Paull,T.T. and Tomkinson,A.E. (2011) Human Mre11/human Rad50/Nbs1 and DNA ligase IIIalpha/XRCC1 protein complexes act together in an alternative nonhomologous end joining pathway. *J. Biol. Chem.*, **286**, 33845–53.
40. Dinkelmann,M., Spehalski,E., Stoneham,T., Buis,J., Wu,Y., Sekiguchi,J.M. and Ferguson,D.O. (2009) Multiple functions of MRN in end-joining pathways during isotype class switching. *Nat. Struct. Mol. Biol.*, **16**, 808–813.
41. Lee,K. and Lee,S.E. (2007) Saccharomyces cerevisiae Sae2- and Tel1-dependent single-strand DNA formation at DNA break promotes microhomology-mediated end joining. *Genetics*, **176**, 2003–14.
42. Ma,J.-L., Kim,E.M., Haber,J.E. and Lee,S.E. (2003) Yeast Mre11 and Rad1 Proteins Define a Ku-Independent Mechanism To Repair Double-Strand Breaks Lacking Overlapping End Sequences. *Mol. Cell. Biol.*, **23**, 8820–8828.
43. Zhang,X. and Paull,T.T. (2005) The Mre11/Rad50/Xrs2 complex and non-homologous end-joining of incompatible ends in S. cerevisiae. *DNA Repair (Amst).*, **4**, 1281–94.
44. Wang,H., Shao,Z., Shi,L.Z., Hwang,P.Y.-H., Truong,L.N., Berns,M.W., Chen,D.J. and Wu,X. (2012) CtIP protein dimerization is critical for its recruitment to chromosomal DNA double-stranded breaks. *J. Biol. Chem.*, **287**, 21471–80.
45. Yun,M.H. and Hiom,K. (2009) CtIP-BRCA1 modulates the choice of DNA double-strand break repair pathway throughout the cell cycle. *Nature*, **459**, 460–463.
46. Audebert,M., Salles,B. and Calsou,P. (2004) Involvement of poly(ADP-ribose) polymerase-1 and XRCC1/DNA ligase III in an alternative route for DNA double-strand breaks rejoining. *J. Biol. Chem.*, **279**, 55117–26.
47. Crespan,E., Czabany,T., Maga,G. and Hübscher,U. (2012) Microhomology-mediated DNA strand annealing and elongation by human DNA polymerases λ and β on normal and repetitive DNA sequences. *Nucleic Acids Res.*, **40**, 5577–90.
48. Liang,L., Deng,L., Nguyen,S.C., Zhao,X., Maulion,C.D., Shao,C. and Tischfield,J.A. (2008)

Human DNA ligases I and III, but not ligase IV, are required for microhomology-mediated end joining of DNA double-strand breaks. *Nucleic Acids Res.*, **36**, 3297–310.
49. Oh,S., Harvey,A., Zimbric,J., Wang,Y., Nguyen,T., Jackson,P.J. and Hendrickson,E.A. (2014) DNA ligase III and DNA ligase IV carry out genetically distinct forms of end joining in human somatic cells. *DNA Repair (Amst).*, **21**, 97–110.
50. Paul,K., Wang,M., Mladenov,E., Bencsik-Theilen,A., Bednar,T., Wu,W., Arakawa,H. and Iliakis,G. (2013) DNA ligases I and III cooperate in alternative non-homologous end-joining in vertebrates. *PLoS One*, **8**, e59505.
51. Costantino,L., Sotiriou,S.K., Rantala,J.K., Magin,S., Mladenov,E., Helleday,T., Haber,J.E., Iliakis,G., Kallioniemi,O.P. and Halazonetis,T.D. (2014) Break-induced replication repair of damaged forks induces genomic duplications in human cells. *Science*, **343**, 88–91.
52. Koole,W., van Schendel,R., Karambelas,A.E., van Heteren,J.T., Okihara,K.L. and Tijsterman,M. (2014) A Polymerase Theta-dependent repair pathway suppresses extensive genomic instability at endogenous G4 DNA sites. *Nat. Commun.*, **5**, 3216.
53. Yu,A.M. and McVey,M. (2010) Synthesis-dependent microhomology-mediated end joining accounts for multiple types of repair junctions. *Nucleic Acids Res.*, **38**, 5706–17.
54. Kent,T., Chandramouly,G., Mcdevitt,S.M., Ozdemir,A.Y. and Pomerantz,R.T. (2015) Mechanism of microhomology-mediated end-joining promoted by human DNA polymerase θ. *Nat. Struct. Mol. Biol.*, **Feb 2**, doi: 10.1038/nsmb.2961.
